# Biomechanics of T Cell Dysfunctions in Chronic Diseases

**DOI:** 10.3389/fimmu.2021.600829

**Published:** 2021-02-25

**Authors:** Sachith D. Gunasinghe, Newton G. Peres, Jesse Goyette, Katharina Gaus

**Affiliations:** ^1^EMBL Australia Node in Single Molecule Science, University of New South Wales, Sydney, NSW, Australia; ^2^ARC Centre of Excellence in Advanced Molecular Imaging, University of New South Wales, Sydney, NSW, Australia

**Keywords:** T cell dysfunction, chronic diseases, infections, cancer, tumor microenvironment, immune receptor landscape, biophysical landscape, biomechanics

## Abstract

Understanding the mechanisms behind T cell dysfunctions during chronic diseases is critical in developing effective immunotherapies. As demonstrated by several animal models and human studies, T cell dysfunctions are induced during chronic diseases, spanning from infections to cancer. Although factors governing the onset and the extent of the functional impairment of T cells can differ during infections and cancer, most dysfunctional phenotypes share common phenotypic traits in their immune receptor and biophysical landscape. Through the latest developments in biophysical techniques applied to explore cell membrane and receptor–ligand dynamics, we are able to dissect and gain further insights into the driving mechanisms behind T cell dysfunctions. These insights may prove useful in developing immunotherapies aimed at reinvigorating our immune system to fight off infections and malignancies more effectively. The recent success with checkpoint inhibitors in treating cancer opens new avenues to develop more effective, targeted immunotherapies. Here, we highlight the studies focused on the transformation of the biophysical landscape during infections and cancer, and how T cell biomechanics shaped the immunopathology associated with chronic diseases.

## Introduction

T cells are at the frontline of immune surveillance, acting against pathogens and malignancies to maintain host homeostasis. Upon recognition of antigenic peptides presented on major histocompatibility complex (MHC) or MHC-like molecules (1, 2), T cells become activated and undergo clonal expansion, resulting in the generation of effector cells that help contain the spread of the disease. During clonal expansion, changes can occur at transcriptional, epigenetic and metabolic levels that enhance the effector functions of T cells (3). Effector T cells produce high amounts of cytokines, including interferon (IFNγ) and tumor necrosis factor (TNFα), and cytoplasmic granules containing granzymes and perforin (4). During antigenic clearance, the majority of effector CD8^+^ T cells follow an apoptotic cell death, but 5–10% of cells differentiate into memory T cells (5). Memory T cells are capable of rapidly executing their effector functions upon re-encounter of the same antigen or pathogen (6, 7). They have a unique transcriptional makeup, which allows them to be distinguished from naïve and effector T cells (8). This unique transcriptional profile shapes the functional characteristics of memory T cells as well as their phenotype to maintain the acquired immunity. Hence, memory T cell differentiation is tightly regulated and any alterations to this process can manifest in various forms of T cell dysfunction (9).

Depending on phenotypic and functional features, T cell dysfunctions can be classified into ignorance, tolerance, exhaustion, anergy or senescence (**Table 1**). These different states of dysfunctions can operate as mechanisms to reduce autoimmunity (ignorance and tolerance), minimise repercussions from inappropriate T cell stimulation (anergy), or simply regulate T cell division (senescence) (9). However, exhaustion brings a unique perspective to the state of T cell dysfunction as it occurs despite physiologically appropriate T cell stimulation. During the transformation of effector T cells into dysfunctional phenotypes, the proliferative capacity along with cytokine production gets reduced (10–12). Moreover, alterations occur at the membrane level transform the immune receptor landscape of T cells (13) and their biophysical properties (14–16). This has ramifications in terms of maintaining optimal T cell responses against pathogens and malignancies. In this review, we highlight recent findings that helped to broaden our understanding on how T cell dysfunctions can reform the immune receptor and biophysical landscape of T cells, and how it can ultimately influence the state of disease progression. We primarily discuss dysfunctional T cell phenotypes in the context of chronic infections and cancer to draw our conclusions.

**Table 1:** Classification of dysfunctional T cells.

T cell dysfunction	Functional and Phenotypic features
Ignorance	Self-reactive T cells that do not sense self-antigens due to physical sequestration or low antigen expression.
Tolerance	Central tolerance accounts for negative selection of thymocytes expressing high-affinity TCRs to self-antigens. Thymocytes which escape negative selection are inactivated by different mechanisms of the peripheral tolerance.
Exhaustion	Persistent antigen stimulation of T cells during chronic diseases (infections or cancer) induce progressive loss of T cell effector functions and lead to exhaustion.
Anergy	A hyporesponsive state of T cells arise in suboptimal costimulatory signal during T cell-antigen recognition.
Senescence	State of non-reversible cell cycle arrest caused by telomere shortening.

## Mechanisms of T Cell Dysfunction in Chronic Diseases

It is conceivable that all T cell dysfunctions stem from alterations occur in the biological process of T cell activation or differentiation. As a result, different dysfunctional T cells share common phenotypic traits, which make the differentiation between T cell dysfunctions subtypes difficult. However, in depth understanding of different molecular mechanisms driving T cell dysfunctions will help to identify signature phenotypic traits to build much clearer and distinguishable profiles for each subtype. Here, we attempt to highlight different mechanisms that drive T cell dysfunctions and the most commonly associated dysfunctional T cells found in chronic diseases.

### T Cell Tolerance and Ignorance

Complete T cell activation requires three signals; first signal provided by TCR-cognitive pMHC interaction, the second is a costimulatory or coinhibitory receptor activation signal provided by APCs, and the third is provided by extracellular cytokines. Of these signals, the second signal becomes crucial in determining the functional outcome of T cell signaling, which may promote T cell effector functions (costimulation) or dampen excessive immune responses (coinhibition) to maintain immunological tolerance (17). Thus, both T cell activation and tolerance are interconnected to tightly regulate and maintain optimal immune responses against foreign antigens while preventing autoimmunity against self-antigens. Failure to maintain immunological tolerance may result in various types of autoimmune diseases. T cell tolerance is primarily enforced by central and peripheral tolerance. Central tolerance operates by eliminating self-reactive T cell through negative selection in the thymus during early stages of T cell development. These T cells express high-avidity TCRs to self-antigens and are mainly eliminated from the system *via* clonal deletion or diverted to differentiate into regulatory T cells (Treg) through thymic negative selection. These elimination mechanisms have been reviewed elsewhere (18). Although majority of self-reactive T cells get screened and eliminated though negative selection in the thymus, this process alone is not sufficient to safeguard against autoimmunity. Self-reactive T cells that escape thymic negative selection are eliminated by peripheral tolerance which acts as the second barrier to maintain immunological tolerance. It was shown that peripheral tolerance is most effective in detecting and eliminating mature T cells that express low-avidity self-reactive TCRs, while central tolerance is effective against eliminating thymocytes expressing high-avidity self-reactive TCRs (19). Peripheral tolerance operates with various mechanisms to inactivate self-reactive T cells that escaped central tolerance. These mechanisms include clonal deletion (20, 21), clonal suppression by Tregs (22–24) and induction of functional non-responsiveness *via* intrinsic cell programming mechanisms (25). It has been suggested that manifestation of a large proportion of autoimmune diseases are linked with the breakdown of peripheral tolerance mechanisms (26–29). In some instances, self-antigens fail to induce negative selection of self-reactive T cells and they become clonally ignorant (30–32). This can be due to low expression of the self-antigen or its physical sequestration at immune-privileged sites like the blood-brain barrier (31). During self-antigen encounter, unlike self-tolerant T cells, self-ignorant T cells remain functional. Most self-ignorant T cells in the periphery are naive, but given the right stimulatory conditions, they can initiate autoimmune responses (33–35).

### T Cell Anergy

Another important state of T cell dysfunction is anergy. The consensus that describe the mechanism behind T cell anergy is based on T cell antigen-stimulation in the absence of the second signal i.e. costimulation, which drives T cells into a hyporesponsive state for an extended period of time (36). Upon re-encounter of the same stimuli with optimal costimulation, anergic T cells fail to proliferate and produce cytokines (36). It has been shown that one of the hallmarks of T cell anergy is the reduced interleukin (IL)-2 production (37). T cell anergy has been broadly classified into clonal anergy and *in vivo* anergy (36). Clonal anergy can be induced in CD4^+^ T cells when stimulated with a strong first signal (TCR-pMHC interaction) and in the absence of the second signal. Low doses of agonist in the presence of costimulation has also been shown to induce clonal anergy (38). *In vivo* anergy also known as adaptive tolerance can occur in the thymus or in the periphery and often associates with naïve T cells during self-antigen stimulation in a costimulation deficient or high coinhibition environment (37, 39). For example, cancer cells and tumor-antigen presenting cells are shown to express high levels of coinhibitory receptor ligands (PD-L1, PD-L2 and many other) with relatively low levels of costimulatory receptor ligands (CD80 and CD86) in the tumor microenvironment to promote T cell anergy (40–43). Despite overlapping functional and phenotypic features, clonal anergy and *in vivo* anergy are driven by distinct molecular mechanisms. For instance, while both anergic phenotypes display either impaired IL-2 production (clonal anergy) or impairment in all TCR-induced cytokine production (*in vivo* anergy) as a key dysfunctional feature during antigen stimulation, only clonal anergy can be rescued by the addition of exogenous IL-2 or by diacylglycerol kinase-α (DGK) inhibitor (44, 45). Clonal and *in vivo* anergy also differ in their signaling defects. TCR-based signaling pathway seems to have impairment in Zap-70 phosphorylation of LAT in the *in vivo* anergy model (37). The signaling pathway of clonal anergy shown to have defects in MAP-kinases activation and mobilisation of NF-κB to the nucleus (46). Anergic phenotypes are associated with a number of autoimmune diseases including human type-1 diabetes (26), systemic lupus erythematosus (47), autoimmune gastritis (48) and myasthenia gravis (49).

### T Cell Senescence

Senescence is recognized as a T cell dysfunction that can play paradoxical roles in adaptive immunity. Based on the triggering mechanisms, T cell senescence can be classified into replicative senescence or premature senescence (50). Replicative senescence occurs as a consequence of telomere shortening after several rounds of cell division, which is associated with the natural aging process (51–53). Hence, an increased number of senescent T cells have been found in the elderly population, which increase their susceptibility for malignancies and chronic diseases (54, 55). However, a number of studies have shown that accumulation of senescent T cells is not limited to the ageing population, but can be found in younger patients with chronic infections and cancer (56–59). The second form of senescence: premature senescence is independent of telomere shortening, and is induced by external factors including cellular stress, particularly oncogenic stress (60, 61). During tumorigenesis, oncogenes become activated and promote uncontrolled cell division, which is commonly observed in many types of cancers. Adversely, a high proliferation rate in cancer cells can become a genetic and metabolic burden which triggers cellular senescence pathways, causing irreversible cell cycle arrest (62, 63). This demonstrates the paradoxical nature of cellular senescence. Moreover, by inducing DNA damage responses, both Tregs and tumor cells can convert T cells to become senescent (64–66). Transformation of effector to senescent T cells dramatically change the immune receptor landscape of T cells. The marked decline of costimulatory receptor expression (CD27 and CD28) (67, 68) is one of the main biomarkers of senescent T cells alongside higher expression of killer cell lectin-like receptor subfamily G member 1 (KLRG-1), Tim-3, CD57 and CD45RA (69–71).

### T Cell Exhaustion

**Figure 1:**
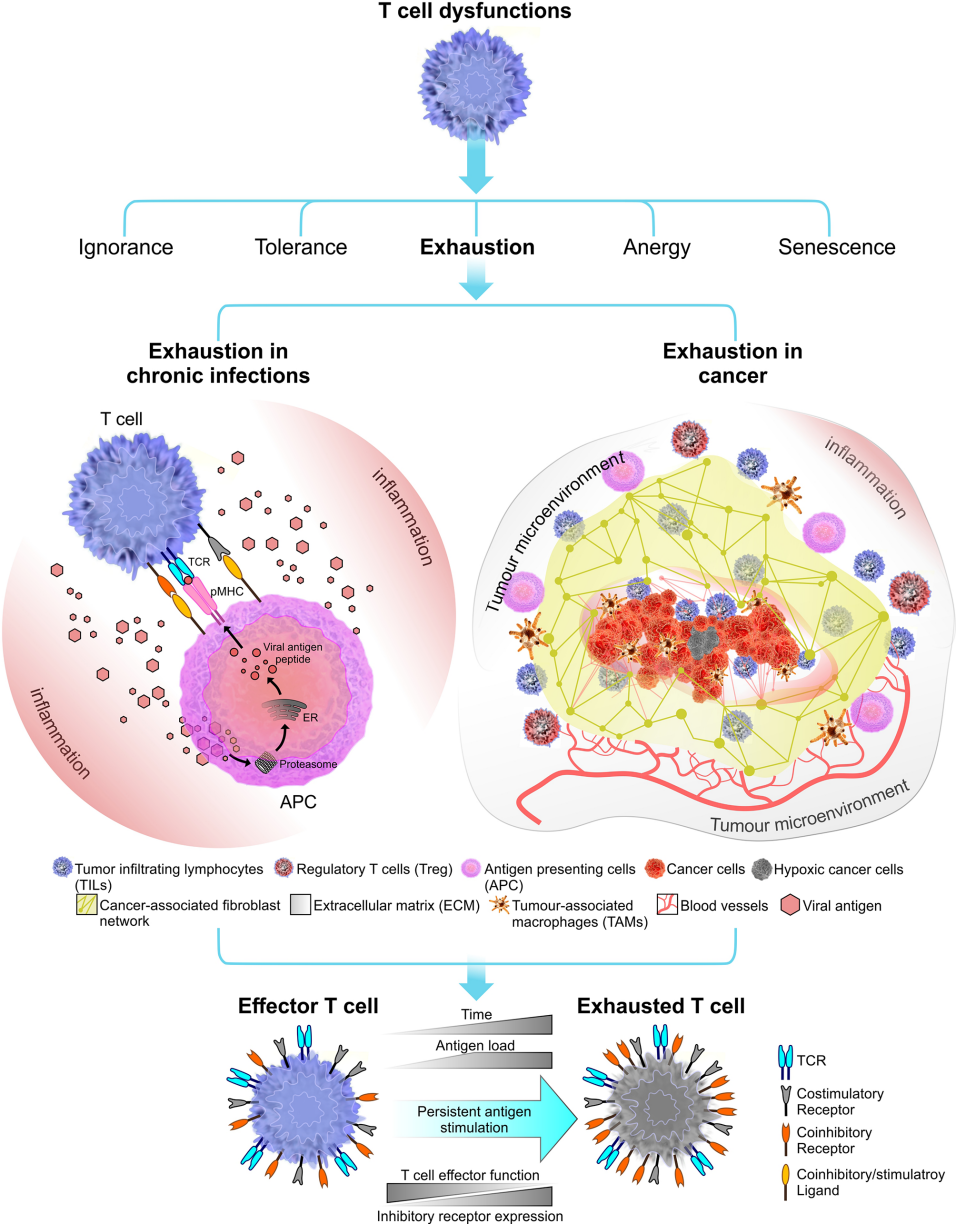
Immune receptor landscape during T cell exhaustion. Exhaustion can be induced by chronic infections (in this instant viral infections) or cancer. Factors that influence the onset and the extent of T cell exhaustion differ in these two exhaustion models. During a chronic infection, pathogen clearance become inefficient, leading to persistent inflammation and chronic antigen stimulation of T cells which results in clonal deletion or exhaustion. In cancer, the immunosuppressive tumor microenvironment plays a crucial role in shaping the outcome of T cell exhaustion. Tumor microenvironment comprised of stroma containing a fibroblast network and a number of immune cells including regulatory T cells (Tregs) and tumor-associated macrophages (TAMs) which together promote tumorigenesis (77). Tumor microenvironment can induce stromal cells to secrete growth factor to promote angiogenesis (i.e. grow new blood vessels that feed the tumor) (78). Overall, T cell exhaustion in both chronic infections and cancer known to have several overlapping functional and phenotypic characteristics. The most common feature is sustained upregulation of inhibitory receptors during the course of the disease.

Since its first characterisation in lymphocytic choriomeningitis virus (LCMV) infection model in mice (72), T cell exhaustion has been the topic of much debate and is implicated in a number of chronic infections (primarily caused by viruses) and cancer (73–76) (**Figure 1**). Over the past few years, the topic of T cell exhaustion has become more relevant as we attempt to uncover the molecular mechanisms behind chronic T cell dysfunctions and develop effective immunotherapies to manage these conditions.

Although driving forces of T cell exhaustion may differ base on different pathological settings, most, if not all proposed mechanisms of T cell exhaustion centres around the three-signal model of T cell activation. Persistent antigen-stimulation, effects of native-regulatory cytokines and immune-suppressive influence of immunoregulatory cells like Tregs are known to promote exhaustion in effector T cells (79). Among these exhaustion inducible factors, persistent antigen-stimulation has been observed across several chronic infection and cancer models in humans and mice (80–82). Accordingly, the dose and the duration of antigen exposure can contribute to the degree of T cell exhaustion. This has been reviewed in later sections of the review.

A key difference has been identified in T cell differentiation during acute and chronic phases of a disease. In the acute phase, T cell mediated antigen clearance comprise of T cell expansion, contraction, and generation of memory T cells. This pattern diverges from the classic differentiation pathway during chronic infections and cancer as a consequence of persistent, higher prevalence of antigens. In this environment, T cells undergo persistent antigen stimulation, which progressively impair their effector functions and drive them to exhaustion. This functional impairment in exhausted T cells, however, does not describe a complete loss of effector functions. As reported by numerous accounts, exhausted T cells still retain some degree of effector functionality having control over the spread of the disease (83–85). Moreover, exhausted T cells share characteristics with memory T cells, which shows their capacity to survive long-term and respond to rechallenge of the antigen (86–89). An important differentiation arises from exhausted T cells having memory characteristics with those do not. Exhausted T cells with memory characteristics have shown to express transcription factor TCF1 (86, 87, 89–91). This subpopulation is responsible for maintaining immune responses during chronic diseases (86, 87, 92) and remains to be critical for the success of anit-PD-1 blockade therapy (87, 93, 94), while TCF1^-^ exhausted T cells fail to provide such responses. In numerous studies TCF^+^ exhausted T cells were described as “stem-like” T cells (87, 89) or “progenitor exhausted” cells (93) and more recently these terms have been unified under “precursor exhausted T cells” (T_PEX_) in contrast to TCF^-^ terminally exhausted effector T cells (T_EX_) (95). Several other transcription factors have been identified to be coexpressed with TCF. Most notably, the T-box transcription factors T-bet and eomesodermin homologue (EOMES) which are known to regulate immune responses during acute and chronic infections (96–98). Their role in maintaining exhaustion phenotypes in both T_PEX_ and T_EX_ needs further investigation. B lymphocyte-induced maturation protein 1 (BLIMP1) is another critical transcription factor required in lymphocyte subset differentiation (99, 100). BLIMP1 suppresses multiple genes linked to T cell memory regulation, therefore, found to be partially expressed in T_PEX_ but not in T_EX_ (87, 91). Other transcription factors relevant in maintaining T cell exhaustion phenotype have been reviewed elsewhere (89, 101–103).

Cytokines play a major role in shaping the outcomes of T cell activation. During the acute phase of infection, proinflammatory cytokines (TNF-α, IL-1β, and IL-8) promote the development of effector T cells to fight off the infection. When the pathogen persists through to the chronic phase, negative regulatory cytokines like IL-10 and TGF-β target different pathways to suppress T cell activation and induce T cell exhaustion. IL-10 is produced by multiple different cells in the tumor microenvironment (TME) including Tregs, tumor-associated macrophages (TAM) and cancer cells (104–106). By inducing PD-L1 expression in dendritic cells, IL-10 promotes T cell exhaustion (107). Downregulation of MHCs, intercellular adhesion molecule 1 (ICAM-1) and costimulatory ligands (CD80 and CD86) on APCs promoted by IL-10 also contribute to immunosuppression (108). Importantly, previous studies have shown that IL-10 plays a role as an anti-inflammatory cytokine by demonstrating its capacity to inhibit or downregulate the production of proinflammatory cytokines (109). It is well known that the surface lattice formed by galectin-glycoprotein can influence membrane remodelling to suppress T cell mediated immune responses (110, 111). Recently, IL-10 was shown to be involved in an immune regulatory loop enhancing N-glycan branching, which heightened galectin-3 binding, thereby decreasing T cell antigen sensitivity (112). Although, IL-10 has been broadly characterized as an immune suppressor, at higher concentrations IL-10 and PEGylated IL-10 (pegilodecakin) has shown to have properties that enhance cytotoxicity and proliferative capacity of tumor-specific CD8^+^ T cells (113–115). The mechanisms underlie this paradoxical nature of IL-10 remains to be investigated. TGF-β is a pleiotropic cytokine and is produced in large amounts in the TME. Generally, TGF-β restrict tumor growth in early stages by inducing the Smad signaling pathway (116). However, in late stages of the cancer, TGF-β has been linked to tumor progression by modulating immune responses most likely through a Smad-independent signaling pathway (117). Given the paradoxical role of TGF-β in cancer, makes it one of the most complex factors to be studied in the TME. Understanding how and when TGF-β switch from tumor suppressor to tumor promoter is being actively investigated.

Immunoregulatory cells including Tregs (CD4 and CD8), myeloid-derived suppressor cells (MDSC) and NK cells are shown to induce T cell exhaustion in effector T cells during chronic infections (118–122). Tregs at the site of the infection or at the TME secrete negative regulatory cytokines, IL-10 and TGF-β, to promote immune suppression (122), thereby limiting anti-pathogen or anti-tumor activity of effector T cells. The exact mechanism of how Treg induce immune suppression to drive effector T cells into exhaustion is still unclear. However, reinvigoration studies of exhausted CD8^+^ T cells associated with chronic LCMV by blocking PD-1 signaling pathway and depleting Tregs simultaneously, suggest a role for Tregs in T cell exhaustion (123).

In summary, although persistent antigen stimulation remains as a key driving force for T cell exhaustion, numerous other factors differentially contribute to the development of exhaustion. In the next section we attempt to highlight two main models of T cell exhaustion found in chronic diseases.

## T Cell Exhaustion Models

From studies reporting common phenotypic characteristics of exhausted T cells, there is an emerging profile that describes T cell exhaustion as a distinct state of cell differentiation. Accordingly, exhaustion phenotypes have been identified in both chronic viral infections and in cancer (**Figure 1**). Although these exhaustion models share common functional features, they differ substantially in some respects.

### T Cell Exhaustion in Chronic Infections

The persistent overload of pathogens during chronic infections leads to persistent antigenic stimulation of T cells. This drive T cells into clonal deletion or exhaustion, both of which lead to reduced pathogen clearance. This is more commonly reported in infections associated with viruses, though T cell exhaustion has also been identified in bacterial and parasitic infections (124, 125). Here, our focus will be on T cell exhaustion during chronic viral infections. During chronic infections, reduced proliferative capacity and low interleukin-2 (IL-2) production (126) are known to be some of the earliest signs of loss of T cell effector functions. At the intermediate state, TNF-α and IFN-γ production are reduced (73). The low cytotoxicity in CD8^+^ T cells is also observed at this stage. Loss of these functional properties occur partially or in severe exhaustion, completely. Finally, these exhausted virus-specific T cells are deleted from the system (72, 74). Hence, a stage-by-stage descent into exhaustion has been observed (10). The level of T cell exhaustion primarily depends on the amount and the strength of antigen stimulation (127). Although the “strength” of stimulus is difficult to define, prolonged exposure to a persistent viral load is an important determinant in the process of exhaustion. For example, higher antigen load with prolong exposure results in severe exhaustion phenotypes seen in LCMV, untreated HBV, and HIV chronic infections (79, 128–130). The role of helper CD4^+^ T cells are also important for promoting effector CD8^+^ T cell functions, thus their low availability has been linked to T cell exhaustion (131, 132). Hence, high viral load and low availability of helper CD4^+^ T cells generally correlates with severe exhaustion phenotype (75). Overall, a number of factors including the viral load, location of viral replication and the immunosuppressive environment, contribute to the level of effector function impairment in T cells during chronic infections.

#### Sustained Upregulation of Inhibitory Receptors

In addition to the gradual loss of effector functions, another classic feature of exhausted T cell is the sustained upregulation of inhibitory receptors (**Figure 1**). These surface expressed inhibitory receptors include programmed cell death protein 1 (PD-1) (133), cytotoxic T lymphocyte associated antigen 4 (CTLA-4), lymphocyte-activation gene 3 (LAG-3), T cell immunoglobulin and mucin-domain containing protein 3 (TIM-3), B and T lymphocyte attenuator (BTLA) and many others (13). In non-pathological settings, the transient expression of inhibitory receptors along with their co-stimulatory counterparts (CD28 and ICOS) serve a key role in maintaining the immunological tolerance. This is readily observed in acute infections where inhibitory receptors contribute to restrain immunopathology after pathogen clearance has been achieved. In fact, upregulation of inhibitory receptors are commonly observed during T cell activation (133–135), although steady-state expression levels may vary depending on the state of cell differentiation (136–138). As pathogen clearance progresses, inhibitory receptors are downregulated and maintained at low levels.

Many inhibitory receptors, including PD-1, can negatively regulate T cell receptor (TCR) signaling *via* immunoreceptor tyrosine-based inhibitory motifs (ITIM) or immunoreceptor tyrosine-based switch motifs (ITSM) found in their cytoplasmic tails (17). Upon binding ligands, ITIM/ITSM domains within the cytoplasmic tails of inhibitory receptors are phosphorylated and recruit Src homology region 2 domain-containing phosphatases (SHP-1 and SHP-2). Overall, PD-1/PD-L1 signaling pathway can regulate exhaustion phenotype by suppressing TCR signaling (139), inducing T cell suppressor genes (140) and by reducing T cell motility (141). TIGIT uses a similar strategy to negatively regulate T cell function (142). However, inhibitory receptors can engage more than one suppressive mechanism to attenuate T cell functions. In contrast to ITIM signaling, LAG-3 is known to function through KIEELE motifs located at its relatively short intracellular tail to negatively regulate cell cycle progression (143). Tim-3 also utilizes non-canonical inhibitory mechanisms that are distinct from, and complementary to, PD-1 (144). High surface expression of Tim-3 often correlates with severely exhausted T cell subsets during chronic infections (145, 146). The inhibitory receptor CTLA-4 functions by outcompeting CD28 stimulatory receptor by binding to their common ligands CD80 or CD86 to suppress T cell functions (147). Uniquely, CTLA-4 can utilize trans-endocytosis; a mechanism of capturing and removing common ligands from the surface of an antigen presenting cell (APC), thus making them unavailable for stimulatory receptor binding (148). All these inhibitory receptors can employ non-overlapping mechanisms of T cell suppression, making their functional role in promoting T cell exhaustion rather diverse and complex.

### T Cell Exhaustion in Cancer

Immunosuppressive factors found in the tumor microenvironment and the tumor-antigen load greatly influence the degree of cancer-mediated T cell exhaustion. Similar to T cell exhaustion in chronic infections, tumor-infiltrating CD8^+^ T cells display attenuated effector functions including impaired cytokine secretion and sustained high surface expression of inhibitory receptors (PD-1, CTLA-4, Tim-3, LAG-3, and others) (76, 149–151). However, exhausted T cells in cancer show subtle differences in their gene expression profiles from infection mediated T cell exhaustion. For example, tumor-specific CD8^+^ T cells derived from a late stage melanoma cancer model showed overexpression of several genes involved in cell cycle regulation, DNA repair and immune responses which was comparatively different from gene expression profiles derived from EBV-specific and CMV-specific exhausted CD8^+^ T cells (152). These differentially expressed genes were related to inhibitory receptors. Accordingly, CD160 and several other inhibitory receptors were not co-expressed in tumor-specific exhausted CD8^+^ T cells compared to virus-specific exhausted CD8^+^ T cells. Some inhibitory receptors like BTLA are upregulated in exhausted tumor-specific CD8^+^ T cells and not in exhausted virus-specific CD8^+^ T cells (76). These distinct gene expression profiles of multiple inhibitory receptors suggest different underlying mechanisms governing receptor upregulation in chronic viral infections and cancer mediated exhaustion. As such, the differential expression of inhibitory receptors may shape the extent of T cell exhaustion in each scenario and provide a molecular signature that will help to diagnose diseases.

#### Tumor Microenvironment

Despite several overlapping functional and phenotypic features found in exhausted T cells induced by chronic viral infections or cancer, the progression of cancer mediated T cell exhaustion is not fully understood. This is partly because of the complexity presented by the tumor microenvironment. The surrounding environment of a developing tumor is comprised of stroma (containing fibroblasts, immune cells, and extracellular matrix) (77), blood vessels, infiltrating inflammatory cells and a number of cells associated with host tissues (**Figure 1**). The cellular environment inside the tumor is not homogenous throughout the cancer (153–155). Hence, the tumor microenvironment (TME) is continuously evolving with tumor progression. Tumor-infiltrating lymphocytes, such as cytotoxic and regulatory T, B and natural killer (NK) cells, associated M2 macrophages (TAM) (156, 157), infiltrating dendritic cells (TIDC) (158) make the TME a battle ground where highly dynamic cellular interactions that take place between the innate and adaptive immune system and the tumor (159). The process of antigen presentation can become impaired inside the TME which may result in incomplete T cell activation (160). Although some T cells are able to infiltrate the tumor, components surrounding the TME including malignant cells, inflammatory cells, stromal cells and cytokines can induce and maintain an immunosuppressive environment that would attenuate T cell effector functions which eventually drive them to exhaustion (153).

#### Immunoediting

With cancer progression, the intense pressure applied by the adaptive immune system and the antigenic heterogeneity of malignant cells allow rare cancer subclones to survive through the elimination phase, equilibrium phase and finally escape from T cell-mediated cytotoxicity (161, 162). In immune-oncology this is known as immunoediting. The theory of immunoediting explains how immunity can play a dual role as a suppressor and as a promoter in cancer (163). Cancer immunoediting is composed of the three phases: elimination, equilibrium and escape (164). In the first two phases, cancer is under control or at a dynamic equilibrium with the immune system, rendering it undetectable *via* clinical methods. As the cancer enters the final phase, it escapes immune surveillance, leading to becoming a clinically detectable progressing tumor.

Immunoediting comprises complex adaptive mechanisms where cancer reduce its immunogenicity to evade recognition and destruction of selected clones (162, 165). Examples of immunoediting are the loss of tumor-associated antigen (TAA) presentation or downregulation of PD-L1 driven by epigenetic changes on cancer cells and abrogated IFNγ—a key regulator of antigen process and presentation—delivered by tumor-infiltrating lymphocytes (TIL) in the TME (166–169), which can lead to incomplete elimination and persistence of adapted tumors becoming clinically evident (169). Insufficient TAA presentation poses a challenge for adaptive cytotoxicity by driving immunological ignorance (170, 171). The poor immunogenicity of transformed cells that escaped recognition can further promote insufficient activation of T cells, evident by unsuccessful immunotherapy treatments in some clinical settings (162). Thus, the selective advantage acquired by evasive cancer cells with impaired T cell responses would follow changes in the immune receptor landscapes on both sides of the immunological synapse. The biophysics of T cell-APC encounter is highly dynamic. The molecular forces at play during these encounters differ greatly in their nature and can trigger unique signaling pathways for cellular decision-making, which has been poorly discussed in the context of T cell dysfunctions.

## Biophysical Landscape of Dysfunctional T Cells

**Figure 2:**
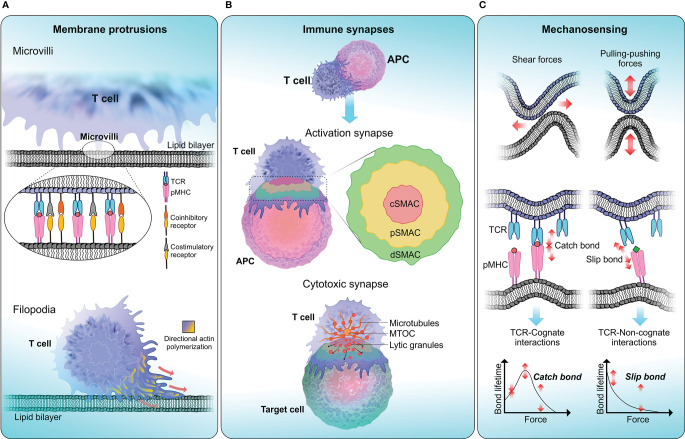
T cell biophysical landscape . **(A)** Membrane protrusions. T cells continuously form actin-rich membrane protrusions known as filopodia or microvilli which help them to sense biophysical properties in the surrounding environment. Microvilli are involved in early T cell activation (173) and where signaling components including TCR and TCR-associated signaling molecules get accumulated (174, 175). The tip of a microvilli is zoomed in to illustrate the accumulation of T cell signaling molecules. Compared to microvilli, filopodium membrane projections are larger in size ranging from 10-40 µm in length in different cells (176). The function of filopodia is broad including crucial roles in cell-cell adhesion and cell migration (177). **(B)** Immune synapses. Formation of immune synapses are important steps in T cell activation and executing T cell effector functions through cytotoxicity. When forming activation synapses, signaling receptors (in T cells) and ligands (in APCs) spatially segregate into a bull’s eye-like structure forming the supra-molecular activation cluster that is separated into central (cSMAC—red), peripheral (pSMAC—yellow) and distal (dSMAC—green) regions, where each zone preferentially recruit different signaling receptors to initiate T cell signaling. Cytotoxic synapses are formed when a CTL encounters a target cell. Cytotoxic synapses differ from activation synapses in the recruitment of lytic granules to the synaptic cleft with the help of Golgi apparatus and the microtubule organising centre (MTOC). Delivery of lytic granules and exocytosis of granule contents are highly depended on calcium influx. **(C)** Mechanosensing. T cells are constantly being subjected to mechanical stresses when undergoing kinapse formation with APCs. TCRs display mechanosensing properties by exerting pulling-pushing and shear forces on pMHC molecules on APCs. When a TCR engage with cognate-antigen peptide, a catch bond is formed, which triggers conformational changes in the TCR-CD3 complex and initiate T cell signaling. Conversely, TCR encounter with a non-cognate-antigen peptide results in a slip bond, where an exponential decay of bond lifetime is observed with increasing force. Slip bonds fail to trigger TCR signaling.

T cell signaling and the cascade of events that follow T cell activation require close encounter of two cell membranes: the plasma membrane of the T cell and the APC. The dynamic and heterogeneous nature of the membrane environment, composed of different types of lipids, receptors and ligands makes the process of T cell-APC conjugation complex to understand. The close contact between the two opposing membranes is accompanied by the formation of unique structural features that promote information transfer through receptor–ligand interactions. The interface between a T cell and an APC is known as the immune synapse and its formation involves spatial redistribution of surface receptors and ligands to facilitate the initiation of immune responses (172). T cells continuously form membrane protrusions known as filopodia or microvilli which help them probe the surface of APCs and to sense biophysical properties in the surrounding environment (**Figure 2A**). Formation of these structural features require extensive membrane remodelling assisted by cytoskeleton rearrangements (178). Moreover, due to relatively high cell motility and relatively slow diffusion rates of engaged receptors and ligands, both cells experience pulling-pushing and shear forces. Recent studies have attempted to quantify these mechanical forces during immune synapse formation (179, 180). It is generally thought that these forces enable T cells to probe the surrounding environment and execute effector functions at optimal levels. Changes to this biophysical landscape could therefore abort T cell responses and disrupt host immune regulatory mechanisms. In this section we highlight the studies that attempt to unravel the link between changes in biophysical properties of the membrane and T cell dysfunction.

### Structure and Functions of Immune Synapses

The immunological synapse is crucial for T cell activation and is sometimes referred to as an activation synapse. Immune receptors, signaling molecules, cytoskeletal components and cell organelles all participate in the formation of the immune synapse (181). In cytotoxic T cells (CTLs), the immune synapse is also the interface in which cytolytic granules are delivered to the target cells and in these cases they have also been referred to as cytotoxic synapses or lytic synapses (182).

#### Immunological Synapses for Activation

When forming activation synapses, key signaling molecules congregate to form a distinct sub-synaptic domain known as the supramolecular activation cluster (SMAC) (**Figure 2B**). The central region of SMAC (cSMAC) primarily contains TCRs and tyrosine kinases which are crucial for the initiation of TCR signaling. The peripheral SMAC (pSMAC) surrounds the cSMAC and contains integrins like LFA-1 which binds to ICAM-1 expressed on APCs, facilitating the adhesion of T cell to APC. Under some circumstances, T cell activation is achieved *via* TCR microclusters and without the need of classic immune synapse formation (183, 184). It has also been proposed that the cSMAC serves as the site for signal termination and receptor recycling (185). One of the main functions of activation synapse is the initiation and amplification of TCR signaling. Upon TCR binding to its cognate pMHC, a cascade of signaling events take place leading to T cell activation, proliferation and execution of effector functions. TCR-pMHC ligation also triggers substantial structural alterations in the membrane (186). These changes permit the recruitment of crucial signaling molecules to the synapse along with accumulation of actin polymers at the pSMAC (187). The surge of F-actin facilitates the formation of membrane structures like lamellipodial which help T cells to spread across APC surface (188).

Overall, optimal T cell-APC contact and effective immune synapse formation is regulated by several cytoskeletal changes. The Vav family of proteins are involved in modulating these cytoskeletal changes at the immune synapse. As shown by previous studies, Vav1 mediates downstream signaling in T cells *via* PLCγ1 and TCR-induced calcium flux (189–191). The absence of Vav1 affects the stability of the TCR signaling clusters and impair both calcium flux and MAP kinase phosphorylation (192). By activating RHO GTPases such as RAC1 and CDC42 (193), Vav1 is implicated in series of events facilitating Wiskott-Aldrich syndrome protein (WASP) and WASP-family verprolin-homologous protein-2 (WAVE2) to activate actin-related protein 2 and 3 (ARP2/3), which leads to polymerisation and accumulation of actin filaments at the immune synapses (194, 195).

#### Cytotoxic Immune Synapses

Cytotoxic synapses are crucial in executing T cell effector functions. One important difference between a cytotoxic synapse and an activation synapse is the recruitment of lytic granules to the synaptic cleft (**Figure 2B**). CTLs exert their cytotoxicity by first binding to the target cell and then releasing lytic granules containing perforin and granzymes *via* exocytosis, and finally detaching from the target cell (196). Similar to activation synapses, attachment to the target cell is primarily mediated by LFA-1 which also aids the formation of SMAC (197). Importantly, the pSMAC has been implicated in stabilising the cytotoxic synapses in CTLs, as the disruption of pSMAC formation results in impaired target cell lysis (198). During CTL mediated target cell lysis, granules containing cytotoxic enzymes (lytic granules) are recruited to the immune synapse with the help of the Golgi apparatus and the microtubule organising centre (MTOC). In NK cells, it was shown that dynein, a cytoskeletal motor protein is responsible for the transport of lytic granules to the MTOC and then MTOC polarises to deliver lytic granules to the synaptic cleft (199). Targeted delivery of lytic granules and exocytosis of granule contents are highly depended on calcium influx (200). Following the detachment from the target cell, CTLs are capable of effectively killing multiple targets sequentially (201).

### Immune Synapse Dysfunctions in Chronic Diseases

Chronic diseases are often associated with the phenotype of T cell exhaustion. The root cause for a number of chronic diseases stems from the inability of T cells to form functional immune synapses with the target cells, leading to impaired T cell activation resulting suboptimal immune responses (202, 203). Understanding the mechanisms that induce impairments in immune synapse formation is an important step in developing effective therapeutics that can reverse T cell exhaustion by restoring effector functions.

Disruption of T cell and target cell contact during T cell activation or T cell-mediated cytotoxicity can impair the formation of functional immune synapses. This can be detrimental in terms of maintaining immunity against pathogens and cellular malignancies. Leukocyte adhesion disorder (LAD) is a classic example of a chronic disease caused by defective expression (LAD type-I) or activation (LAD type-III) of cell adhesion molecules, primarily β-2 integrins like LFA-1 (204). Since LFA-1 plays a crucial role in the assembly of immune synapses, LAD patients often have recurrent bacterial infections due to their compromised immune system (205). Similarly, defects in WASP family of proteins directly affect actin mediated cytoskeletal rearrangement during immune synapse formation. This was shown in Wiskott-Aldrich Syndrome where CTLs lose their cytotoxicity (206). In a rare type of non-Hodgkin lymphoma known as anaplastic large cell lymphoma (ALCL), it has been shown that WASP and WASP-interacting-protein (WIP) are expressed in low amounts (207). In WASP knockout (KO) mice, fast onset of tumor growth has been observed (208). In the same study, the metastatic rate of B16 melanoma was shown to be higher, indicating an overall loss of T cell tolerance towards the cancer. However, somewhat contradictory observations were made in mouse breast carcinoma, where the metastatic spread was decreased in the absence of WASP (209), suggesting differing roles for WASP in cancer progression depending on the cancer model. Interestingly, addition of exogenous IL-2 was able to rescue the cytotoxicity of WASP KO NK cells by restoring their ability to form immune synapses (210, 211). In fact, IL-2 treatment is commonly used as an immunotherapy treatment in the attempt to promote T cell proliferation and restore or enhance T cell effector functions (212). WASP is one of many proteins in which irregular protein expression can lead to immune dysfunctions because of cytoskeletal organisation defects during immune synapse formation. A growing number of putative proteins including Dock8, RAC2, RHOH, CORO1A, ACTB and many others have been implicated in modulating actin-dependent cytoskeleton organisation to promote efficient T cell activation. Their individual functions have been reviewed elsewhere (213).

Failure to deliver lytic granules to the synaptic cleft leave CTLs with impaired cytotoxicity and reduced pathogen clearance. The continual stimulation from the innate immune system together with dysfunctional adaptive immune responses can result in systemic inflammation which is detrimental to the host homeostasis. This is readily observed in herpes viral infections, particularly Epstein–Barr virus (EBV) and cytomegalovirus (CMV). These viruses with their lifelong latency in the host may result in persistent antigenic-stimulation mediated by the innate immune system (214). This has been linked to hemophagocytic lymphohistiocytosis (HLH), a life-threatening syndrome presented with attenuated killing capacity of T cells and NK cells (215).

In cancer models, much of the evidence for defects in the formation of immune synapses comes from haematological malignancies (15, 216, 217). For instance, one report demonstrated that CD8^+^ and CD4^+^ T cells are unable to form proper immune synapses with chronic lymphocytic leukemia (CLL) cells, which hindered anti-tumor activity (15). The authors show that when a healthy T cell encounters CLL-B cells, F-actin polymerisation was suppressed and there was impaired recruitment of key adhesion and signaling molecules to the immune synapse of the T cells (15). These observations agree with other cancer models where tumor-infiltrating lymphocytes (TIL) showed similar defects in actin polymerisation (218, 219). The exact mechanism behind tumor-induced immune synapse defects in T cells is not yet clear. However, immuno-modulatory drugs such as lenalidomide are shown to be effective in reversing actin polymerisation defects in patients with follicular lymphoma (216).

Human immunodeficiency virus type-1 (HIV-1) also induces signaling dysfunctions in CD4^+^ T cells as a part of its viral pathogenesis. The abundantly expressed viral protein Nef plays a central role in impairing the immune synapse formation in HIV-1 infected T cells (220). Nef achieves this by hijacking the host membrane protein trafficking machinery to promote the spread of infection (221). HIV-1 infected T cells showed poor cell spreading, suggesting a Nef-dependent inhibition of actin polymerisation. In parallel, a reduced recruitment of TCR-CD3 complex, Lck and other actin polymerisation-related proteins to the immune synapses was observed (222). Interestingly, Nef sequesters Lck away from TCR-CD3 complex, in both the presence or absence of CD4, and slows down TCR internalisation (220), thereby arresting TCR recycling and downregulation following TCR-pMHC ligation. This leads to accumulation of TCRs on the cell surface, resulting in T cell hyperactivation which is readily observed in untreated HIV-infection (223, 224). Previous reports also show that Nef has downstream effects on transcription factors like NFAT and NF- κB which are important to execute T cell mediated immune responses (225, 226).

From T cell-APC conjugation to T cell activation and cytolytic granule trafficking to targeted cytotoxicity, these chronic diseases highlight the importance of each stage in immune synapse formation for the execution of optimal T cell immune responses.

### T Cell Mechanosensing

The initial contact between T cell and APC demonstrated by TCR binding to its cognate antigen-peptide triggers downstream signaling events that would activate T cells to efficiently execute their effector functions. However, the outcome of TCR signaling is largely impacted by mechanical forces applied to the TCR-pMHC complex (227, 228). Essentially, exogenous forces applied to TCR-pMHC interactions are transmitted into the cell as biochemical signals through mechanotransduction, the process which describes how physical perturbation experienced by receptors are translated into chemical signals (229). Conversely, biochemical signals generated by the cell is being translated into mechanical forces that are exerted on the surrounding environment.

Attempts to *ex vivo* activate and expand T cells utilising soluble anti-CD3 antibodies have largely failed (228, 230–232). TCR triggering *ex vivo* can be achieved by immobilising CD3 complex activating antibodies on rigid surfaces such as beads or tissue culture plates, as evidenced by the increased Ca^2+^ influx and phosphorylation of ZAP70 in T cells (233, 234). One explanation for why surface attached antibodies induce activation but those in solution do not is that surface association mimics mechanical forces created by the movements of synaptic membranes on TCR-pMHC complexes and suggests that TCR signaling cannot be initiated unless pulling-pushing stresses or shear forces are applied to the complex (**Figure 2C**) (234, 235).

Early studies showed that mechanical forces at the piconewton (pN) range applied through pMHC-coated beads were enough to induce Ca^2+^ influx and ERK phosphorylation in T cells (236). However, when similar forces were applied to CD28, CD62L, or ICAM-2 no significant increase in Ca^2+^ influx was observed (234). Since these mechanical forces involving antigen recognition by T cells operate at pN range, it poses technical challenges when elucidating them in biological systems. Recently, using biomembrane force probe (BFP) a number of studies have shown the threshold for cognate TCR-pMHC interaction to be at the scale of ~10 pN (237, 238). In addition to TCRs, other mechanosensors such as Piezo1 contributes to optimal T cell signaling (232). However, the extent of Piezo1 involvement in TCR signaling and T cell responses is yet to be elucidated. As the mechanical forces applied on TCR-pMHC require some level of rigidity from both biological supports (i.e. membranes), the stiffness of APC is expected to influence T cell responses. Stimulating substrates with anti-CD3 and anti-CD28 antibodies on supports with relatively high rigidity drive greater productions of IFN-γ, TNF-α and IL-2, up-regulation of activation markers and proliferative capacity compared with softer substrates (180, 228, 239–241). Furthermore, CTLs increase the stiffness of APC by stretching the synaptic region to modulate the speed of the perforin pore formation and consequently promote faster target cell lysis (242). These studies highlight the importance of fine tuning the rigidity of stimulating cultures for optimal T cell response which has implications in adoptive T cell immunotherapies (239, 240, 243, 244). One may also question whether subtle mechanical changes in TCR-pMHC affinity can trigger specific signal transduction pathways and affect downstream T cell responses.

Forces applied on TCR-pMHC complex can affect their bond lifetime in unexpected ways. For instance, catch bonds, where pulling forces applied to the bond, increases its bond lifetime (245), have been described for several receptor–ligand interactions (**Figure 2C**) (246, 247) and recently also for TCR-agonistic peptide MHC interactions (237, 238, 248). In contrast, antagonistic peptides form slip bonds characterized by short lifetimes (**Figure 2C**) (237). Independent reports using BFP (237, 238, 249, 250) and optical tweezers (248) on cell systems that express transgenic TCRs and cell-free experiments also using optical tweezers (248, 251) have demonstrated that while agonistic-peptides can increase TCR-pMHC binding lifetime, antagonistic-peptides tends to reduce it. These studies further demonstrated that catch bonds reach their maximum lifetime under a mechanical force in the range of ~10 pN and the lifetime of slip bonds decreases exponentially with increased mechanical force.

Another crucial aspect of T cell mechanosensing is to understand how TCR and cognate-pMHC binding events get translated into biochemical signals in T cells that are specific to the antigenic peptide. One theory suggests a conformational change of the TCR-CD3 complex during pMHC binding that would dislodge and release the cytoplasmic tails of CD3 from the inner leaflet of the plasma membrane. This would expose immunoreceptor tyrosine-based activation motifs (ITAMs) to get phosphorylated by tyrosine kinases Lck and Fyn. Accordingly, a TCR specific antigenic peptide would expose the cytoplasmic tails of CD3 for a longer period and permit more efficient phosphorylation by tyrosine kinases to generate a stronger signal to transduce (252, 253). A theory based on kinetic segregation model explains a local disruption of the kinase-phosphatase balance during TCR-pMHC binding is sufficient to generate a productive signal as TCRs get phosphorylated by the segregation of phosphatase like CD45 (254, 255). Here, catch bonds (formed with cognate ligands) or slip bonds (formed with non-cognate ligands) formed during TCR-pMHC interactions (**Figure 2C**) may generate differential segregation patterns that would then be translated to a strong or weak signal, respectively (250).

In summary, regulating mechanosensing capacity of TCR is crucial to recognise and translate mechanical cues into cell signals during T cell activation and in execution of immune responses.

#### T Cell Mechanosensing in Chronic Diseases

T cells constantly patrol and migrate to different tissue compartments in search of cognate-antigens. This tissue migration involves continuous changes in T cell morphology driven by actin polymerisation which impose considerable mechanical force at the cellular level. During the contact between T cell and APC, the role played by mechanical forces in mediating immune responses are now becoming clear. The highly dynamic interactions between the extracellular matrix (ECM) and actin cytoskeleton is directly linked with translating mechanical cues from the environment into cell signals. Substrate stiffness is a mechanical cue that is implied to regulate number of cellular functions including proliferation, migration and differentiation (256–258). T cells are exposed to a range of substrate stiffnesses during their lifespan as stiffness values change substantially in different cells that T cells encounter. For example, while skeletal muscles have a stiffness in the range of ~10 kPa (259), elastic modulus of human bones may vary from 7–25 GPa (260).

A number of studies reported that reduced stiffness in cancer cells as a mechanism of promoting their growth independent of ECM stiffness (261, 262). Since optimal T cell responses require surfaces or biological membranes with relatively high rigidity, by reducing surface stiffness, cancer cells can effectively evade immune detection and subsequent cytotoxicity. Concurrently, a local increase in ECM stiffness is associated with disease progression (263). It has been reported that cancer cells are able to modify their surrounding ECM stiffness in order to promote metastasis (264). In fact, ECM associated adhesion proteins are known to play a vital role in different stages of cancer metastasis which overall influence the invasiveness of a cancer (265). In some cases, cancer cells are shown to synthesise their own ECM proteins to promote metastasis (266). Additionally, ECM stiffness influence the outcome of desmoplastic response (i.e. pervasive growth of dense collagen stroma around a tumor) associated with tumors (267). For instant, desmoplasia in pancreatic and breast cancer promote tumor progression and results in poor prognosis (268, 269). In mammary tumors, lysyl oxidase enzyme is linked to remodelling and increasing ECM stiffness as the inhibition of this enzyme reduced tissue stiffening and delayed tumor progression (270). Several studies have demonstrated a correlation between collagen density, a primary component of the ECM, and the infiltrative capacity of T cells into tumor islets (271–273). Densely packed collagen fibres are suggested to obstruct T cell entry into the tumor microenvironment, and overall reduce their proliferative and cytotoxic capacity (273). Recently, a study using confocal microscopy coupled with optical tweezers was able to track changes in biophysical properties of cancer cells in a multicellular 3D breast cancer model (274). The study was able to identify a stiffness gradient decreasing outward from the core of the growing tumor, suggesting cancer cells with softer biophysical characteristics are likely to be located at the periphery of the tumor. Moreover, this also implies that T cell mediated cytotoxicity become less efficient at the edge of the tumor, thereby increasing the invasiveness of the tumor at the periphery. Whether modulation of cell stiffness is a reliant mechanism for immune evasion during chronic infections is yet to be determined. Overall, understanding T cell responses to mechanical cues such as substrate stiffness may become crucial to understand the biophysical landscape of exhausted T cells.

### TCR Diversity in Immune Responses

In adaptive immunity, the engagement of TCR with pMHC molecules plays a pivotal role in shaping the overall immune responses against foreign pathogens, malignancies and allergens. When TCRs recognise and bind to their cognate antigen, it triggers intracellular signaling pathways that activate the expression of multiple genes linked to several effector functions in T cells. Hence, the quality and magnitude of T cell effector functions are linked to the strength and quality of TCR-pMHC interactions. Primarily, the strength of these interactions are measured by TCR affinity to its antigen (275). T cell signaling is a complex function that involves the affinity of TCR-pMHC interactions, coreceptor binding and co-stimulatory and co-inhibitory signal integration (276), but in general, affinity of TCR-pMHC can predict the sensitivity of a T cell to a specific antigen (277). TCR affinity also dictates selective polyclonal expansion of antigen-specific T cells during immune responses.

Rearrangement within the variable regions of the TCR and thymic selection generates an immune repertoire of T cells with differing antigen specificities. During an infection, the expansion of T cell clones specific to a small number of immunodominant antigens can skew the immune repertoire (278). This form of TCR bias can be influenced by multiple factors ranging from thymic selection to initial immune response to an antigen (278). Overall, the adaptive immune system is shown to maintain a diversified population of antigen-specific T cell clones with varying affinities which possess the capacity to clonally expand and form memory T cells. In some cases a bias T cell clonal expansion, either towards high or low affinity has been observed (279, 280). During the acute phase of an infection, early models of clonal selection have shown that antigen-specific T cells with higher affinity are selectively expanded from the polyclonal T cell population to mediate immune responses and proceed to become memory T cells to retain acquired immunity (281–283). This selective enrichment of antigen-specific high-affinity clones has been described as a form of affinity maturation of T cells (284). During persistent antigen exposure, however, the profile of memory T cell clones shifts towards a low-affinity repertoire (16, 279, 285). Hence, distinct affinity profiles for antigen-specific T cells are generated and maintained during acute and chronic phase of an infection. Elucidating the underline mechanisms behind this differential clonal expansion under different phases of antigen exposure proven to be beneficial in developing therapeutic interventions aimed at restoring T cell immune responses in chronic diseases.

#### Measuring TCR Affinity

**Figure 3:**
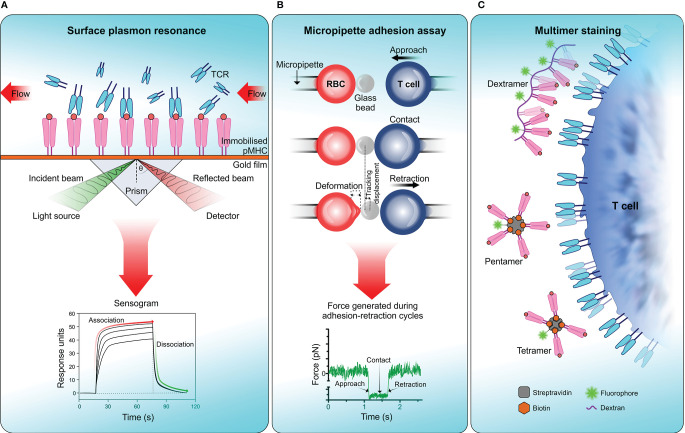
Measuring TCR affinity . **(A)** Surface plasmon resonance (SPR). SPR measures the equilibrium dissociation constant (*K_D_*) of TCR-pMHC interactions in which pMHC is immobilized on a sensor surface and TCR molecules are injected in a continuous flow. Binding of TCR to pMHC results in a change of mass on the sensor surface and is recorded in a sensogram which is then used to calculate *K_D_*. **(B)** Micropipette adhesion assay. This technique uses two probes, one that is stationary which contains a red blood cell (RBC) attached to a functionalized glass bead to act as the adhesion force transducer and a mobile force probe bearing a T cell coupled to a piezotranslator. During adhesion-retraction cycles carried out by the mobile probe, the deformation of the RBC, displacement of the glass bead and the force generated in each cycle is recorded. **(C)** Multimer staining. This technique enhances the binding avidity of TCR-pMHC by increasing the valency of the interaction, results in more stable multimeric TCR-pMHC complexes for efficient labelling and detection. To date numerous forms of pMHC multimers have been reported which includes tetramers, pentamers, octamers, and dextramers (289).

TCR affinity to its cognate antigen-MHC complex is generally reported as the ratio of *k_off_* and *k_on_* rates, *K_D_*: the equilibrium dissociation constant. In simple terms TCR-pMHC interactions with low *K_D_* values (i.e. high affinity interactions) are typically associated with longer binding dwell times. Due to rapid dissociation (high *k_off_*) of TCR-pMHC complex, it is often difficult to determine the affinity of TCR-pMHC interactions using conventional kinetic measurements. Generally, TCR-pMHC affinity is in the scale of micromolar range (1 to 300 µM), albeit with considerable variability (275, 286). Among several approaches which have been useful in characterising TCR-pMHC binding affinity, surface plasmon resonance (SPR), two-dimensional micropipette adhesion frequency assay (2D‐MP) (287, 288) and pMHC multimer-binding have demonstrated their wide applicability in physiological settings (**Figure 3**).

The usage of SPR to determine TCR-pMHC affinity dates back three decades (290, 291). SPR measures a signal that correlates to a change in mass on a sensor-surface (i.e. sensor chip) where the binding partner, in this case pMHC molecules are immobilized on the senor-surface and TCR molecules flown over to bind (**Figure 3A**). SPR offers much lower sample requirement and versatility over earlier techniques like isothermal titration calorimetry (ITC), but with some drawbacks which have been reviewed elsewhere (292, 293). While SPR provides the means of directly measuring TCR-pMHC binding affinity, in physiological settings, the TCR is also attached to a surface where several other receptor–ligand interactions would take place simultaneously. Moreover, SPR would not account for the forces introduced with bystander TCRs, auxiliary receptors, cell adhesion molecules and membrane fluctuations which overall can modify TCR-pMHC binding affinity. Hence, measuring functional TCR-pMHC binding affinity in native cell membrane environment would be more physiologically relevant (288).

When predicting TCR-pMHC affinity in the context of T cell-APC interactions, the 2D micropipette adhesion assay (2D-MP) technique has proven to be useful. In 2D-MP, a human red blood cell (RBC) decorated with the ligand of interest acts as a sensor for measuring adhesion kinetics with the cell of interest expressing the cognate receptor (**Figure 3B**) (294). A micromanipulation device is used to bring these two cells into close proximity, in a tightly controlled environment enabling receptor–ligand binding. These binding events are captured as the degree of deformation of RBC membrane when the T cell is pulled away. These adhesion cycles were repeated and then translated into a binding curve which allows the calculation of binding kinetics for a given receptor–ligand interaction in two-dimensional space. By measuring affinity in the native membrane environment of a receptor, this method provides more physiologically relevant binding kinetics that have more applicability in cell biology. A modified version of 2D-MP known as the fluorescence biomembrane force probe (fBFP) uses osmolarity adjusted human red blood cell attached to a functionalized glass bead to act as the adhesion force transducer (**Figure 3B**) (237). This technique combined with single-molecule force spectroscopy and fluorescence microscopy enables the measurement of singular receptor–ligand binding event kinetics. Both 2D-MP and fBFP have measured much faster off-rates for TCR-pMHC interactions [30–8,300 fold faster (287)] than reported by 3D kinetic measurements derived from SPR. It should be noted that rapid 2D off-rates obtained for TCR-pMHC do not necessarily correlate with the rapid off-rates in 3D, indicating kinetics of TCR-pMHC interactions including off-rates and antigen-peptide affinity differ substantially from 2D to 3D space. A major drawback of these techniques is their requirement for highly specialized equipment to measure cellular level kinetics. This limits their usage in predicting population level kinetics during T cell-APC interactions.

Multimers of pMHC are the most commonly used method in identifying antigen-specific T cells from a polyclonal population. Due to their low affinity, monomeric pMHC are ineffective as a labelling probe in detecting antigen-specific T cell clones from a pool of other T cells. Multimer technology overcomes this by increasing the valency of TCR-pMHC interaction by multimerizing pMHC complexes to increasing avidity, which results in more stable multimeric TCR-pMHC complexes for efficient labelling and detection (295). The pMHC multimers can be in the form of tetramers, pentamers or octamers (**Figure 3C**) (296, 297). Recently, multimer labelling has been shown to introduce biases towards detection of high-affinity TCR-pMHC interactions and underestimation of interactions with low-affinity. This may distort the overall view of antigen-specific T cell diversity in a polyclonal population (298). Therefore, multimer binding intensity does not necessarily correlate with the functional responses produced by antigen-specific T cell population (299, 300).

It is evident that each affinity measurement technique in isolation overlooks the clonal diversity of T cell immune repertoire which is crucial to understand the full extent of immune responses during a disease. When combined, these techniques would resolve the shortcomings of affinity biases in predicting antigen-specific polyclonal T cell diversity.

#### T Cell Affinity Repertoire in Chronic Diseases

TCR affinity and signaling strength in response to a specific antigen sets the threshold for clonal selection to execute immune responses. Based on the premise that high-affinity TCR-pMHC interactions leads to efficient T cell activation, high-affinity T cell clones have a selective advantage over other clonotypes in mediating primary and secondary immune responses during the acute phase of a disease (281, 283, 301). This observed lack of affinity diversity becomes reduced when the disease progresses into a chronic phase. Mounting evidence suggest that this enhanced diversity in T cell clonal affinity is due to the recruitment of low-affinity TCR expressing T cells in the immune repertoire (280, 302–304). This distribution pattern of affinity clones of antigen-specific T cells can differ between disease models, and how this diversity is maintained to produce life-long immune responses is still under investigation. Moreover, screening affinity diversity in the T cell immune repertoire is challenging as current detection methods are suboptimal in identifying the full breadth of clonal diversity. Excluding low-affinity clones from the measured T cell repertoire will underestimate the full capacity of functional responses exerted by the immune system during chronic diseases.

A number of studies have demonstrated the effectiveness of low-affinity antigen-specific T cell clones in mediating immune responses, combating infections and preventing tumor progression similar to high-affinity counterparts (279, 280, 300, 305). Based on CD4^+^ T cell responses to six different LCMV-antigens, Martinez et al. reported limited correlation between TCR affinity and dominance in clonal expansion (280). Moreover, both high and low-affinity T cell clones possess similar proliferative capacity (306, 307) and phenotypic characteristics (302) which all together challenge the prerequisite of high-affinity TCR-pMHC interaction dominance in driving clonal expansion and mediating immune responses. However, the strength of TCR-pMHC ligation may determine the magnitude of clonal expansion and the onset of contraction phase. This was demonstrated in *Listeria monocytogenes* infection model using altered peptide ligands with varying affinities (302). Despite the observed similarities in rapid proliferation rates in low and high-affinity antigen-specific T cell clones, Zehn et al. showed that weaker ligand interactions lead to early onset of contraction of T cell proliferation (302). In the same study, early appearance of low-affinity T cell clones in the blood stream after antigen-stimulation suggest a role for TCR-pMHC affinity in modulating the kinetics of T cell migration. Moreover, the reduced memory T cell expansion during successive challenge of a weak ligand/antigen indicates a correlation with the strength of recall stimulus and memory T cell responses.

Despite numerous attempts undertaken to predict the affinity diversity of T cells in different disease models, most clonal diversity observations of immune responsive T cells come from unrelated models of acute and chronic diseases. It would be highly relevant to demonstrate the evolutionary trajectory of antigen-specific T cell affinity using longitudinal observations during acute and chronic phase of the same disease model. Using two LCMV infection models, Andargachew et al. showed that the overall affinity diversity of CD4^+^ T cell clones were similarly maintained throughout acute and chronic antigen exposure along with effector and memory T cells showing similar affinity distribution patterns in both phases (308). Their study accompanied 2D-affinity kinetic measurements derived from 2D-MP assay. During the transition of effector to early memory CD4^+^ T cells, both acute and chronic LCMV infection showed an increased functional avidity. However, the half-maximal effective concentration (EC_50_) for IFN-γ and IL-2 production was much lower for acute-LCMV CD4^+^ T cells compared to chronic exhausted CD4^+^ T cells, which indicates a higher antigen sensitivity and functional avidity for CD4^+^ T cells in the acute phase of LCMV infection. This study also drew parallels between chronic and acute-LCMV with their selective recruitment of low-affinity T cell clones into the immune repertoire.

More recently, a longitudinal study used memory T cell inflation to illustrate the evolutionary trajectory of cytomegalovirus (CMV)-specific CD8^+^ effector memory T cell affinity during acute and chronic phase antigen exposure (16). T cell inflation is described as the atypical accumulation of memory T cells in blood and peripheral tissues in response to persistent low-level antigen exposure (309). Using both human and mouse CMV models, Schober et al. analyzed TCR affinity distribution among the CD8^+^ T cell population. The TCR-pMHC dissociation rate (*k_off_*) measurements obtained from real-time fluorescence microscopy (310) conclusively demonstrated that T cells with lower TCR affinity were enriched in the inflationary CD8^+^ T cell pool compared to the acute phase. Moreover, in-depth analysis of CMV-specific TCR repertoire obtained from the mouse model showed clones under-represented in the acute phase of the infection (medium to low-affinity clones) were recruited at higher proportions to the immune repertoire at later stages *via* clonal succession (311). The authors also suggest that recruitment of low-affinity T cell clones compensated the loss of functional avidity provided by high-affinity clones which become senescent at late stages of the infection. This form of “reversed-affinity maturation” could be an important adaptation of the immune system to maintain life-long effective T cell responses against persistent viral infections which particularly exhibit low antigen expression levels. In the long-run, selective expansion of low-affinity T cell clones may provide an effective strategy in generating lasting pathogen control along with reduced immunopathology.

Whether the above mechanisms prove to be effective in regulating clonal diversity in T cell exhaustion related chronic diseases remains to be explored. T cells with high-functional avidity often exhibit exhaustion phenotypes under high levels of antigen exposure (312, 313). This can increase the probability of pathogen escape from the immune system, leaving low-avidity clones of antigen-specific T cells to take up the task of pathogen clearance or maintain a life-long host-pathogen equilibrium with reduced immunopathology. It should be noted that low-avidity T cell clones can also become functionally exhausted as reported by several tumor models (314, 315).

The affinity repertoire of effector T cells during cancer is less well understood than in chronic infections. Previous studies suggest that TILs with high-avidity are more likely show exhaustion markers albeit having superior control in eliminating tumor cells compared to their low-avidity counterparts (312, 316, 317). A growing number of studies have recognized a distinct role for low-avidity TILs in tumor clearance. Studies have shown low-affinity TCR interactions with tumor antigens activate tumor-specific T cells in a similar manner to high-affinity TCR interactions with the tumor antigen (317, 318). Moreover, with prolong exposure to the tumor, both clonotypes showed exhaustion markers including sustained upregulation of inhibitory receptors, with higher degree of exhaustion observed for high-avidity tumor-specific T cell clones (317). Another study using adoptive transfer of OT-I (high-affinity) and OT-3 (low-affinity) transgenic tumor-specific CD8^+^ T cells was able to demonstrate that OT-3 T cells were able to mediate tumor regression in pancreas with minimum autoimmunity, contrast to OT-I T cells which in addition to the rapid eradication of the tumor, caused autoimmune diabetes in the mouse model (314). These studies suggest a necessary role for low-avidity tumor-specific T cells in anti-cancer immune responses. Thus, elucidating various mechanism underlying the expansion of T cell affinity repertoire during cancer progression is important to understand cancer immune surveillance and more complex immune-oncology concepts like immunoediting. Immunoediting of malignant cells can generate slightly variable neoantigen presented to the existing TCR repertoire in the TME causing decreases in the overall TCR affinities and T cell-cytolytic responses, and consequently tumor evasion (319–321).

#### Other Factors Influencing T Cell Affinity Diversity

Numerous other internal and external factors including the expression level of co-stimulatory/inhibitory receptors in T cells, co-stimulatory/inhibitory ligands on APC, and the dose and density of antigen presentation by APCs play a key role in shaping the functional avidity of antigen-specific T cell immune repertoire. For example, the CD27/CD70 mediated co-stimulation in T cells has been shown to lower the threshold of TCR activation to respond to low-affinity antigens, which promotes to generate a higher degree of memory T cell clonal diversity (322). Conversely, higher expression of B7-1 along with ICAM-1 and LFA-3 are linked to selectively enriching T cell clones with high functional avidity (323, 324). *De novo* expression of B7-1 by anti-myeloma cellular vaccines improves cytotoxicity and helper-dependent memory formation of subdominant CD8^+^ T cell clones by avoiding tolerogenic effects (320). The antigen density presented by APCs during T cell priming and during infections has been shown to influence the functional avidity of immune responsive T cell populations (325). For instance, higher antigen density on APCs can compensate for low-affinity TCR-pMHC interactions. In human melanoma model it was demonstrated that low antigen doses presented by dendritic cells (DCs) produce melan-A-specific CD8^+^ T cells with high functional avidity which had lower dependence on CD8 coreceptors (326). B cells in infection models like Friend virus (FV) are linked to efficient priming and subsequent expansion of T cells with low functional avidity, overall diversifying CD4^+^ T cell immune repertoire (327). Moreover, the degree of B cell activation correlates with B cell mediated clonal expansion of low-avidity CD4^+^ T cells. Indeed, antigen specific B cells have long been speculated to drive clonal diversity in CD4^+^ T cells (328).

## Conclusion

Dysfunctional T cells are distinct from effector and memory T cells based on their functionality, metabolic activity, and epigenetic makeup. Recent findings strengthen the link between dysfunctional T cells and the progression of chronic diseases, thus, unravelling potential mechanisms behind the functional impairment of T cells with changes to its immune receptor and biophysical landscape during disease progression. In T cell exhaustion, the sustained upregulation of inhibitory receptors becomes a key feature that modifies the immune receptor landscape of T cells. Hence, these receptors have become primary targets in developing checkpoint blockade therapies aimed at restoring effector functions in non-responsive T cells. Although this has shown much clinical success in managing the progression of chronic diseases, there remains to be several limitations which hinder its wide applicability. Acquired resistance is one of the emerging challenges faced by checkpoint blockade therapy and may be overcome by combinatorial therapeutic strategies. The effectiveness of these therapeutic approaches in rescuing terminally exhausted T cells remains to be explored.

Apart from alterations in immune receptors expression profile, understanding changes to the cellular physiology of T cells during disease progression has become increasingly relevant to elucidate factors that promote T cell dysfunctions. Throughout their lifetime T cells are subjected to a myriad of mechanical forces experienced during cell migration, cell–cell interactions or exerted by the surrounding ECM. It is now becoming clear that these forces have important roles in T cell activation and the resultant effector functions, and may also play a central role in T cell dysfunctions. The concept of mechanotransduction as a mechanism of regulating cell behavior and function is not new (329), however, to understand this process at a subcellular level by means of measuring these infinitesimal forces require hypersensitive tools. As highlighted in this review, we have discussed the application of biophysical tools that can measure the strength of TCR-pMHC interactions as a proxy to predict the quality and magnitude of T cell mediated immune responses. Other techniques, including traction force microscopy (TFM) (180, 330), micro-pillar array detectors (mPADs) (331) and DNA-based molecular tension sensors (332, 333) have demonstrated to be useful in measuring mechanical forces experienced by TCRs *in vivo*. Importantly, DNA-based tension gauge tether was able to map mechanical forces during T cell activation modulated on a nanoparticle surface (332). This technique has been useful in determining T cell force threshold to distinguish functionally relevant mechanical forces from those do not trigger T cell activation, thus, providing a “fidelity checkpoint” for antigen discrimination (332). Utilization of these techniques to exploit single-molecule biomechanics of immune receptors may become useful in elucidating more complex cellular interactions faced by T cells such as found in the TME. Further, with the aid of these advanced biophysical tools, it is possible to develop new class of immunotherapies that aims to revamp T cell effector functions by recalibrating the mechanical force threshold of T cells to trigger more effective anti-tumor immune responses against the stiffness gradient of a growing tumor.

Lately, the usage of engineered chimeric antigen receptor (CAR) T cells as an effective immunotherapy has been useful in treating several cancer models (334–336). Importantly, previously observed non-classical immune synapses formation in CAR T cells correlates with the rapid recruitment of lytic granules to the synaptic cleft and killing the target cells much faster than classic CTLs (184). These unique functional features can be utilized to improve immunotherapy treatments against solid tumors. However, the potency of CAR T cells has been limited by several factors including T cell exhaustion (337, 338). The early constructs of CARs possessed affinities in the range of nano molar scale, rendering high-affinity interactions between CAR and antigens (339). These interactions are much stronger than physiologically relevant affinities displayed by TCR and pMHC, leading to off-target toxicities. This emphasises the role of affinity modulation in immunotherapy, which also becomes useful in designing prophylactic vaccine strategies to develop lasting immunity against pathogens. Immunotherapies aimed at treating patients in the acute phase of an infection should utilize the proliferative capacity of high-avidity T cell clones to achieve pathogen clearance. Accordingly, when an acute infection exacerbates into the chronic phase where pathogen clearance becomes inefficient, immunotherapies should make use of low-avidity clones to promote lasting host–pathogen equilibrium which delivers minimal immunopathology.

The importance of T cell biomechanics and how they differ between T cell subtypes needs further investigation. So far, much of the evidence of biomechanical influence in mediating T cell immune responses comes from expansion of clonal avidity of antigen-specific T cells during infections or cancer. Overall, in depth understanding of the biophysical properties behind mediating optimal immune reposes may help to identify broader principles governing T cell dysfunctions in chronic diseases and present new and improved avenues to develop clinical interventions in the future.

## Author Contributions

SG and NP reviewed the relevant literature and wrote the manuscript. JG and KG provided critical feedback and helped shape the manuscript. All authors contributed to the article and approved the submitted version.

## Funding

The authors would like to acknowledge funding from the Australian Research Council (ARC) (CE140100011 to KG), the National Health and Medical Research Council of Australia (APP1155162 to KG), and Cancer Council New South Wales (APP1128488 to KG).

## Conflict of Interest

The authors declare that the research was conducted in the absence of any commercial or financial relationships that could be construed as a potential conflict of interest.

## References

[1] Rock KL Reits E Neefjes J. Present Yourself! By MHC Class I and MHC Class II Molecules. Trends Immunol (2016) 37:724–37. 10.1016/j.it.2016.08.010 PMC5159193 27614798

[2] Natarajan K Jiang J May NA Mage MG Boyd LF McShan AC. The role of molecular flexibility in antigen presentation and T cell receptor-mediated signaling. Front Immunol (2018) 9:1657. 10.3389/fimmu.2018.01657 30065727 PMC6056622

[3] Masopust D Schenkel JM. The integration of T cell migration, differentiation and function. Nat Rev Immunol (2013) 13:309–20. 10.1038/nri3442 23598650

[4] Cox MA Harrington LE Zajac AJ. Cytokines and the inception of CD8 T cell responses. Trends Immunol (2011) 32:180–6. 10.1016/j.it.2011.01.004 PMC3074938 21371940

[5] Kalia V Sarkar S Ahmed R. CD8 T-cell memory differentiation during acute and chronic viral infections. Adv Exp Med Biol (2010) 684:79–95. 10.1007/978-1-4419-6451-9_7 20795542

[6] Wherry EJ Ahmed R. Memory CD8 T-Cell Differentiation during Viral Infection. J Virol (2004) 78:5535–45. 10.1128/jvi.78.11.5535-5545.2004 PMC415833 15140950

[7] Williams MA Bevan MJ. Effector and Memory CTL Differentiation. Annu Rev Immunol (2006) 25:171–92. 10.1146/annurev.immunol.25.022106.141548 17129182

[8] Kaech SM Hemby S Kersh E Ahmed R. Molecular and functional profiling of memory CD8 T cell differentiation. Cell (2002) 111:837–51. 10.1016/S0092-8674(02)01139-X 12526810

[9] Schietinger A Greenberg PD. Tolerance and exhaustion: Defining mechanisms of T cell dysfunction. Trends Immunol (2014) 35:51–60. 10.1016/j.it.2013.10.001 24210163 PMC3946600

[10] Wherry EJ. T cell exhaustion. Nat Immunol (2011) 12:492–9. 10.1038/ni.2035 21739672

[11] Saeidi A Zandi K Cheok YY Saeidi H Wong WF Lee CYQ. Shankar EM. T-cell exhaustion in chronic infections: Reversing the state of exhaustion and reinvigorating optimal protective immune responses. Front Immunol (2018) 9:2569. 10.3389/fimmu.2018.02569 30473697 PMC6237934

[12] Wang C Singer M Anderson AC. Molecular Dissection of CD8+ T-Cell Dysfunction. Trends Immunol (2017) 38:567–76. 10.1016/j.it.2017.05.008 PMC5759349 28662970

[13] Blackburn SD Shin H Haining WN Zou T Workman CJ Polley A. Coregulation of CD8+ T cell exhaustion by multiple inhibitory receptors during chronic viral infection. Nat Immunol (2009) 10:29–37. 10.1038/ni.1679 19043418 PMC2605166

[14] Duong MN Erdes E Hebeisen M Rufer N Chronic TCR-MHC. (self)-interactions limit the functional potential of TCR affinity-increased CD8 T lymphocytes. J Immunother Cancer (2019) 7:284. 10.1186/s40425-019-0773-z 31690351 PMC6833194

[15] Ramsay AG Johnson AJ Lee AM Gorgün G Le DR Blum W. Chronic lymphocytic leukemia T cells show impaired immunological synapse formation that can be reversed with an immunomodulating drug. J Clin Invest (2008) 118:2427–37. 10.1172/JC135017 PMC2423865 18551193

[16] Schober K Voit F Grassmann S Müller TR Eggert J Jarosch S. Reverse TCR repertoire evolution toward dominant low-affinity clones during chronic CMV infection. Nat Immunol (2020) 21:434–41. 10.1038/s41590-020-0628-2 32205883

[17] Chen L Flies DB. Molecular mechanisms of T cell co-stimulation and co-inhibition. Nat Rev Immunol (2013) 13:227–42. 10.1038/nri3405 PMC3786574 23470321

[18] Takaba H Takayanagi H. The Mechanisms of T Cell Selection in the Thymus. Trends Immunol (2017) 38:805–16. 10.1016/j.it.2017.07.010 28830733

[19] Liu GY Fairchild PJ Smith RM Prowle JR Kioussis D Wraith DC. Low avidity recognition of self-antigen by T cells permits escape from central tolerance. Immunity (1995) 3:407–15. 10.1016/1074-7613(95)90170-1 7584132

[20] Hernandez J Aung S Redmond WL Sherman LA. Phenotypic and functional analysis of CD8+ T cells undergoing peripheral deletion in response to cross-presentation of self-antigen. J Exp Med (2001) 194:707–17. 10.1084/jem.194.6.707 PMC2195957 11560988

[21] Kurts C Kosaka H Carbone FR Miller JFAP Heath WR. Class I-restricted cross-presentation of exogenous self-antigens leads to deletion of autoreactive CD8+ T cells. J Exp Med (1997) 186:239–45. 10.1084/jem.186.2.239 PMC2198972 9221753

[22] Wing K Sakaguchi S. Regulatory T cells exert checks and balances on self tolerance and autoimmunity. Nat Immunol (2010) 11:7–13. 10.1038/ni.1818 20016504

[23] Sakaguchi S. Naturally arising Foxp3-expressing CD25+ CD4+ regulatory T cells in immunological tolerance to self and non-self. Nat Immunol (2005) 6:345–52. 10.1038/ni1178 15785760

[24] Izcue A Coombes JL Powrie F. Regulatory lymphocytes and intestinal inflammation. Annu Rev Immunol (2009) 27:313–38. 10.1146/annurev.immunol.021908.132657 19302043

[25] Schietinger A Delrow JJ Basom RS Blattman JN Greenberg PD. Rescued tolerant CD8 T cells are preprogrammed to reestablish thetolerant state. Science (2012) (80-):723–7. 10.1126/science.1214277 PMC3754789 22267581

[26] Burrack AL Martinov T Fife BT. T cell-mediated beta cell destruction: Autoimmunity and alloimmunity in the context of type 1 diabetes. Front Endocrinol (Lausanne) (2017) 8:343. 10.3389/fendo.2017.00343 29259578 PMC5723426

[27] Skapenko A Leipe J Lipsky PE Schulze-Koops H. The role of the T cell in autoimmune inflammation. Arthritis Res Ther (2005) 7:S4–14. 10.1186/ar1505 15833146 PMC2833981

[28] Weiner HL. Multiple sclerosis is an inflammatory T-cell-mediated autoimmune disease. Arch Neurol (2004) 61:1613–5. 10.1001/archneur.61.10.1613 15477521

[29] Jabri B Sollid LM. T Cells in Celiac Disease. J Immunol (2017) 198:3005–14. 10.4049/jimmunol.1601693 PMC5426360 28373482

[30] Parish IA Heath WR. Too dangerous to ignore: Self-tolerance and the control of ignorant autoreactive T cells. Immunol Cell Biol (2008) 86:146–52. 10.1038/sj.icb.7100161 18227854

[31] Ohashi PS Oehen S Buerki K Pircher H Ohashi CT Odermatt B. Ablation of “tolerance” and induction of diabetes by virus infection in viral antigen transgenic mice. Cell (1991) 65:305–17. 10.1016/0092-8674(91)90164-T 1901764

[32] Kurts C Sutherland RM Davey G Li M Lew AM Blanas E. (1999) CD8 T cell ignorance or tolerance to islet antigens depends on antigen dose. Proc Natl Acad Sci USA 96:12703–7. 10.1073/pnas.96.22.12703 PMC23058 10535986

[33] Oldstone MBA Nerenberg M Southern P Price J Lewicki H. Virus infection triggers insulin-dependent diabetes mellitus in a transgenic model: Role of anti-self (virus) immune response. Cell (1991) 65:319–31. 10.1016/0092-8674(91)90165-U 1901765

[34] Millar DG Garza KM Odermatt B Elford AR Ono N Li Z. Hsp70 promotes antigen-presenting cell function and converts T-cell tolerance to autoimmunity in vivo. Nat Med (2003) 9:1469–76. 10.1038/nm962 14625545

[35] Ramanathan S Dubois S Chen X-L Leblanc C Ohashi PS Ilangumaran S. Exposure to IL-15 and IL-21 Enables Autoreactive CD8 T Cells To Respond to Weak Antigens and Cause Disease in a Mouse Model of Autoimmune Diabetes. J Immunol (2011) 186:5131–41. 10.4049/jimmunol.1001221 21430227

[36] Schwartz RH. T cell anergy. Annu Rev Immunol (2003) 21:305–334. 10.1146/annurev.immunol.21.120601.141110 12471050

[37] Choi S Schwartz RH. Molecular mechanisms for adaptive tolerance and other T cell anergy models. Semin Immunol (2007) 19:140–52. 10.1016/j.smim.2007.02.005 PMC2045643 17400472

[38] Mirshahidi S Huang C Sadegh-Nasseri S. Anergy in peripheral memory CD4+ T cells induced by low avidity engagement of T cell receptor. J Exp Med (2001) 194:719–31. 10.1084/jem.194.6.719 PMC2195956 11560989

[39] Jenkins MK Schwartz RH. Antigen presentation by chemically modified splenocytes induces antigen-specific T cell unresponsiveness in vitro and in vivo. J Exp Med (1987) 165:302–19. 10.1084/jem.165.2.302 PMC2188516 3029267

[40] Blank C Brown I Peterson AC Spiotto M Iwai Y Honjo T. PD-L1/B7H-1 Inhibits the Effector Phase of Tumor Rejection by T Cell Receptor (TCR) Transgenic CD8+ T Cells. Cancer Res (2004) 64:1140–5. 10.1158/0008-5472.CAN-03-3259 14871849

[41] Zou W Chen L. Inhibitory B7-family molecules in the tumour microenvironment. Nat Rev Immunol (2008) 8:467–77. 10.1038/nri2326 18500231

[42] Kryczek I Zou L Rodriguez P Zhu G Wei S Mottram P. B7-H4 expression identifies a novel suppressive macrophage population in human ovarian carcinoma. J Exp Med (2006) 203:871–81. 10.1084/jem.20050930 PMC2118300 16606666

[43] Curiel TJ Wei S Dong H Alvarez X Cheng P Mottram P. Blockade of B7-H1 improves myeloid dendritic cell-mediated antitumor immunity. Nat Med (2003) 9:562–67. 10.1038/nm863 12704383

[44] Boussiotis VA Barber DL Nakarai T Freeman GJ Gribben JG Bernstein GM. Prevention of T cell anergy by signaling through the γc chainof the IL-2 receptor. Science (1994) (80-):1039–42. 10.1126/science.7973657 7973657

[45] Zha Y Marks R Ho AW Peterson AC Janardhan S Brown I. T cell anergy is reversed by active Ras and is regulated by diacylglycerol kinase-α. Nat Immunol (2006) 7:1166–73. 10.1038/ni1394 17028589

[46] Chiodetti L Choi S Barber DL Schwartz RH. Adaptive Tolerance and Clonal Anergy Are Distinct Biochemical States. J Immunol (2006) 176:2279–91. 10.4049/jimmunol.176.4.2279 16455984

[47] Moulton VR Tsokos GC. Abnormalities of T cell signaling in systemic lupus erythematosus. Arthritis Res Ther (2011) 13:207. 10.1186/ar3251 PMC3132009 21457530

[48] Harakal J Rival C Qiao H Tung KS. Regulatory T Cells Control Th2-Dominant Murine Autoimmune Gastritis. J Immunol (2016) 197:27–41. 10.4049/jimmunol.1502344 27259856 PMC4912916

[49] Reim J McIntosh K Martin S Daniel BD. Specific immunotherapeutic strategy for myasthenia gravis: targeted antigen-presenting cells. J Neuroimmunol (1992) 41:61–70. 10.1016/0165-5728(92)90196-R 1460093

[50] Zhao Y Shao Q Peng G. Exhaustion and senescence: two crucial dysfunctional states of T cells in the tumor microenvironment. Cell Mol Immunol (2020) 17:27–35. 10.1038/s41423-019-0344-8 31853000 PMC6952436

[51] Collado M Blasco MA Serrano M. Cellular Senescence in Cancer and Aging. Cell (2007) 99:1047–78. 10.1016/j.cell.2007.07.003 17662938

[52] Collado M Serrano M. Senescence in tumours: Evidence from mice and humans. Nat Rev Cancer (2010) 10:51–7. 10.1038/nrc2772 PMC3672965 20029423

[53] Kasakovski D Xu L Li Y. T cell senescence and CAR-T cell exhaustion in hematological malignancies. J Hematol Oncol (2018) 11:91. 10.1186/s13045-018-0629-x 29973238 PMC6032767

[54] Chou JP Effros RB. T Cell Replicative Senescence in Human Aging. Curr Pharm Des (2013) 19:1680–98. 10.2174/138161213805219711 PMC3749774 23061726

[55] Weng N-P Akbar AN Goronzy J. CD28- T cells: their role in the age-associated decline of immune function. Trends Immunol (2009) 30:306–12. 10.1016/j.it.2009.03.013 PMC2801888 19540809

[56] Tsukishiro T Donnenberg AD Whiteside TL. Rapid turnover of the CD8+CD28- T-cell subset of effector cells in the circulation of patients with head and neck cancer. Cancer Immunol Immunother (2003) 52:599–607. 10.1007/s00262-003-0395-6 12827303 PMC11032778

[57] Appay V Nixon DF Donahoe SM Gillespie GMA Dong T King A. HIV-specific CD8+ T cells produce antiviral cytokines but are impaired in cytolytic function. J Exp Med (2000) 192:63–75. 10.1084/jem.192.1.63 10880527 PMC1887711

[58] Montes CL Chapoval AI Nelson J Orhue V Zhang X Schulze DH. Tumor-induced senescent T cells with suppressor function: A potential form of tumor immune evasion. Cancer Res (2008) 68:870–9. 10.1158/0008-5472.CAN-07-2282 18245489

[59] Wolfram RM Budinsky AC Brodowicz T Kubista M Köstler WJ Kichler-Lakomy C. Defective antigen presentation resulting from impaired expression of costimulatory molecules in breast cancer. Int J Cancer (2000) 88:239–44. 10.1002/1097-0215(20001015)88:2<239::AID-IJC15>3.0.CO;2-Z 11004675

[60] Zeng S Shen W Liu L. Senescence and Cancer. Cancer Transl Med (2018) 4:70–4. 10.4103/ctm.ctm_22_18 PMC6372122 30766922

[61] Liu X-L Ding J Meng L-H. Oncogene-induced senescence: a double edged sword in cancer. Acta Pharmacol Sin (2018) 39:1553–8. 10.1038/aps.2017.198 PMC6289471 29620049

[62] Sarkisian CJ Keister BA Stairs DB Boxer RB Moody SE Chodosh LA. Dose-dependent oncogene-induced senescence in vivo and its evasion during mammary tumorigenesis. Nat Cell Biol (2007) 9:493–505. 10.1038/ncb1567 17450133

[63] Courtois-Cox S Jones SL Cichowski K. Many roads lead to oncogene-induced senescence. Oncogene (2008) 27:2801–9. 10.1038/sj.onc.1210950 18193093

[64] Liu X Mo W Ye J Li L Zhang Y Hsueh EC. Regulatory T cells trigger effector T cell DNA damage and senescence caused by metabolic competition. Nat Commun (2018) 9:249. 10.1038/s41467-017-02689-5 PMC5770447 29339767

[65] Ye J Peng G. Controlling T cell senescence in the tumor microenvironment for tumor immunotherapy. Oncoimmunology (2015) 4:e994398. 10.4161/2162402X.2014.994398 PMC4404789 25949919

[66] Ye J Ma C Hsueh EC Dou J Mo W Liu S. TLR 8 signaling enhances tumor immunity by preventing tumor-induced T-cell senescence. EMBO Mol Med (2014) 6:1294–311. 10.15252/emmm.201403918 PMC4287933 25231413

[67] Ye J Huang X Hsueh EC Zhang Q Ma C Zhang Y. Human regulatory T cells induce T-lymphocyte senescence. Blood (2012) 120:2021–31. 10.1182/blood-2012-03-416040 PMC3437594 22723548

[68] Vallejo AN. CD28 extinction in human T cells: Altered functions and the program of T-cell senescence. Immunol Rev (2005) 205:158–69. 10.1111/j.0105-2896.2005.00256.x 15882352

[69] Li H Wu K Tao K Chen L Zheng Q Lu X. Tim-3/galectin-9 signaling pathway mediates T-cell dysfunction and predicts poor prognosis in patients with hepatitis B virus-associated hepatocellular carcinoma. Hepatology (2012) 56:1342–51. 10.1002/hep.25777 22505239

[70] Brenchley JM Karandikar NJ Betts MR Ambrozak DR Hill BJ Crotty LE. Expression of CD57 defines replicative senescence and antigen-induced apoptotic death of CD8+ T cells. Blood (2003) 101:2711–20. 10.1182/blood-2002-07-2103 12433688

[71] Heffner M Fearon DT. Loss of T cell receptor-induced Bmi-1 in the KLRG1+ senescent CD8+ T lymphocyte. Proc Natl Acad Sci USA (2007) 104:13414–9. 10.1073/pnas.0706040104 PMC1941641 17686974

[72] Moskophidis D Lechner F Pircher H Zinkernagel RM. Virus persistence in acutely infected immunocompetent mice by exhaustion of antiviral cytotoxic effector T cells. Nature (1993) 362:758–61. 10.1038/362758a0 8469287

[73] Wherry EJ Blattman JN Murali-Krishna K van der Most R Ahmed R. Viral Persistence Alters CD8 T-Cell Immunodominance and Tissue Distribution and Results in Distinct Stages of Functional Impairment. J Virol (2003) 77:4911–27. 10.1128/jvi.77.8.4911-4927.2003 PMC152117 12663797

[74] Zajac AJ Blattman JN Murali-Krishna K Sourdive DJD Suresh M Altman JD. Viral immune evasion due to persistence of activated T cells without effector function. J Exp Med (1998) 188:2205–13. 10.1084/jem.188.12.2205 PMC2212420 9858507

[75] McLane LM Abdel-Hakeem MS Wherry EJ. CD8 T Cell Exhaustion During Chronic Viral Infection and Cancer. Annu Rev Immunol (2019) 37:457–95. 10.1146/annurev-immunol-041015-055318 30676822

[76] Fourcade J Sun Z Pagliano O Guillaume P Luescher IF Sander C. CD8 + T cells specific for tumor antigens can be rendered dysfunctional by the tumor microenvironment through upregulation of the inhibitory receptors BTLA and PD-1. Cancer Res (2012) 72:887–96. 10.1158/0008-5472.CAN-11-2637 PMC3288235 22205715

[77] Bremnes RM Dønnem T Al-Saad S Al-Shibli K Andersen S Sirera R. The Role of Tumor Stroma in Cancer Progression and Prognosis. J Thorac Oncol (2011) 6(1):209–17. 10.1097/JTO.0b013e3181f8a1bd 21107292

[78] Watnick RS. The role of the tumor microenvironment in regulating angiogenesis. Cold Spring Harb Perspect Med (2012) 2(12):a006676. 10.1101/cshperspect.a006676 23209177 PMC3543072

[79] Wherry EJ Kurachi M. Molecular and cellular insights into T cell exhaustion. Nat Rev Immunol (2015) 15(8):486–99. 10.1038/nri3862 PMC4889009 26205583

[80] Shin H Blackburn SD Blattman JN Wherry EJ. Viral antigen and extensive division maintain virus-specific CD8 T cells during chronic infection. J Exp Med (2007) 204(4):941–9. 10.1084/jem.20061937 PMC2118542 17420267

[81] Wherry EJ Ha SJ Kaech SM Haining WN Sarkar S Kalia V. Molecular Signature of CD8+ T Cell Exhaustion during Chronic Viral Infection. Immunity (2007) 27(4):670–84. 10.1016/j.immuni.2007.09.006 17950003

[82] Schietinger A Philip M Krisnawan VE Chiu EY Delrow JJ Basom RS. Tumor-Specific T Cell Dysfunction Is a Dynamic Antigen-Driven Differentiation Program Initiated Early during Tumorigenesis. Immunity (2016) 45(2):389–401. 10.1016/j.immuni.2016.07.011 27521269 PMC5119632

[83] Speiser DE Utzschneider DT Oberle SG Münz C Romero P Zehn D. T cell differentiation in chronic infection and cancer: Functional adaptation or exhaustion? Nat Rev Immunol (2014) 14(11):768–74. 10.1038/nri3740 25257362

[84] Philip M Schietinger A. Heterogeneity and fate choice: T cell exhaustion in cancer and chronic infections. Curr Opin Immunol (2019) 58:98–103. 10.1016/j.coi.2019.04.014 31181510 PMC7608527

[85] Li H van der Leun AM Yofe I Lubling Y Gelbard-Solodkin D van Akkooi ACJ. Dysfunctional CD8 T Cells Form a Proliferative, Dynamically Regulated Compartment within Human Melanoma. Cell (2019) 176(4):775–89.e18. 10.1016/j.cell.2018.11.043 30595452 PMC7253294

[86] Utzschneider DT Charmoy M Chennupati V Pousse L Ferreira DP Calderon-Copete S. T Cell Factor 1-Expressing Memory-like CD8+ T Cells Sustain the Immune Response to Chronic Viral Infections. Immunity (2016) 45(2):415–27. 10.1016/j.immuni.2016.07.021 27533016

[87] Im SJ Hashimoto M Gerner MY Lee J Kissick HT Burger MC. Defining CD8+ T cells that provide the proliferative burst after PD-1 therapy. Nature (2016) 537(7620):417–21. 10.1038/nature19330 PMC5297183 27501248

[88] Utzschneider DT Legat A Fuertes Marraco SA Carrié L Luescher I Speiser DE. T cells maintain an exhausted phenotype after antigen withdrawal and population reexpansion. Nat Immunol (2013) 14(6):603–10. 10.1038/ni.2606 23644506

[89] Wu T Ji Y Ashley Moseman E Xu HC Manglani M Kirby M. The TCF1-Bcl6 axis counteracts type I interferon to repress exhaustion and maintain T cell stemness. Sci Immunol (2016) 1(6):eaai8593. 10.1126/sciimmunol.aai8593 28018990 PMC5179228

[90] Brummelman J Mazza EMC Alvisi G Colombo FS Grilli A Mikulak J. High-dimensional single cell analysis identifies stemlike cytotoxic CD8+T cells infiltrating human tumors. J Exp Med (2018) 215(10):2520–35. 10.1084/JEM.20180684 PMC6170179 30154266

[91] Leong YA Chen Y Ong HS Wu D Man K Deleage C. CXCR5+ follicular cytotoxic T cells control viral infection in B cell follicles. Nat Immunol (2016) 17(10):1187–96. 10.1038/ni.3543 27487330

[92] He R Hou S Liu C Zhang A Bai Q Han M. Follicular CXCR5-expressing CD8+ T cells curtail chronic viral infection. Nature (2016) 537(7620):412–28. 10.1038/nature19317 27501245

[93] Miller BC Sen DR Al Abosy R Bi K Virkud YV LaFleur MW. Subsets of exhausted CD8+ T cells differentially mediate tumor control and respond to checkpoint blockade. Nat Immunol (2019) 20(3):326–36. 10.1038/s41590-019-0312-6 PMC6673650 30778252

[94] Siddiqui I Schaeuble K Chennupati V Fuertes Marraco SA Calderon-Copete S Pais Ferreira D. Intratumoral Tcf1 + PD-1 + CD8 + T Cells with Stem-like Properties Promote Tumor Control in Response to Vaccination and Checkpoint Blockade Immunotherapy. Immunity (2019) 50(1):195–211.e10. 10.1016/j.immuni.2018.12.021 30635237

[95] Kallies A Zehn D Utzschneider DT. Precursor exhausted T cells: key to successful immunotherapy? Nat Rev Immunol (2020) 20(2):128–36. 10.1038/s41577-019-0223-7 31591533

[96] Intlekofer AM Takemoto N Wherry EJ Longworth SA Northrup JT Palanivel VR. Effector and memory CD8+ T cell fate coupled by T-bet and eomesodermin. Nat Immunol (2005) 6(12):1236–44. 10.1038/ni1268 16273099

[97] Joshi NS Cui W Chandele A Lee HK Urso DR Hagman J. Inflammation Directs Memory Precursor and Short-Lived Effector CD8+ T Cell Fates via the Graded Expression of T-bet Transcription Factor. Immunity (2007) 27(2):281–95. 10.1016/j.immuni.2007.07.010 PMC2034442 17723218

[98] Pearce EL Mullen AC Martins GA Krawczyk CM Hutchins AS Zediak VP. Control of Effector CD8+ T Cell Function by the Transcription FactorEomesodermin. Science (2003) (80-):1041–3. 10.1126/science.1090148 14605368

[99] Kallies A Nutt SL. Terminal differentiation of lymphocytes depends on Blimp-1. Curr Opin Immunol (2007) 19(2):156–62. 10.1016/j.coi.2007.01.003 17291741

[100] Kallies A Xin A Belz GT Nutt SL. Blimp-1 Transcription Factor Is Required for the Differentiation of Effector CD8+ T Cells and Memory Responses. Immunity (2009) 31(2):283–95. 10.1016/j.immuni.2009.06.021 19664942

[101] Khan O Giles JR McDonald S Manne S Ngiow SF Patel KP. TOX transcriptionally and epigenetically programs CD8+ T cell exhaustion. Nature (2019) 571(7764):211–8. 10.1038/s41586-019-1325-x PMC6713202 31207603

[102] Delpoux A Michelini RH Verma S Lai CY Omilusik KD Utzschneider DT. Continuous activity of Foxo1 is required to prevent anergy and maintain the memory state of CD8 + T cells. J Exp Med (2018) 215(2):575–94. 10.1084/jem.20170697 PMC5789410 29282254

[103] Seo H Chen J González-Avalos E Samaniego-Castruita D Das A Wang YH. TOX and TOX2 transcription factors cooperate with NR4A transcription factors to impose CD8+ T cell exhaustion. Proc Natl Acad Sci USA (2019) 116(25):12410–5. 10.1073/pnas.1905675116 PMC6589758 31152140

[104] Qi L Yu H Zhang Y Zhao D Lv P Zhong Y. IL-10 secreted by M2 macrophage promoted tumorigenesis through interaction with JAK2 in glioma. Oncotarget (2016) 7(44):71673–85. 10.18632/oncotarget.12317 PMC5342110 27765933

[105] Blackburn SD Wherry EJ. IL-10, T cell exhaustion and viral persistence. Trends Microbiol (2007) 15(4):143–6. 10.1016/j.tim.2007.02.006 17336072

[106] Gastl GA Abrams JS Nanus DM Oosterkamp R Silver J Liu F. Interleukin-10 production by human carcinoma cell lines and its relationship to interleukin-6 expression. Int J Cancer (1993) 55(1):96–101. 10.1002/ijc.2910550118 8344757

[107] Kim Y-J Park S-J Broxmeyer HE. Phagocytosis, a Potential Mechanism for Myeloid-Derived Suppressor Cell Regulation of CD8 + T Cell Function Mediated through Programmed Cell Death-1 and Programmed Cell Death-1 Ligand Interaction. J Immunol (2011) 187(5):2291–301. 10.4049/jimmunol.1002650 PMC3159723 21795591

[108] Asadullah K Sterry W Volk HD. Interleukin-10 therapy - Review of a new approach. Pharmacol Rev (2003) 55(2):241–69. 10.1124/pr.55.2.4 12773629

[109] Kessler B Rinchai D Kewcharoenwong C Nithichanon A Biggart R Hawrylowicz CM. Interleukin 10 inhibits pro-inflammatory cytokine responses and killing of Burkholderia pseudomallei. Sci Rep (2017) 7:42791. 10.1038/srep42791 28216665 PMC5316963

[110] Rabinovich GA Liu FT Hirashima M Anderson A. An emerging role for galectins in tuning the immune response: Lessons from experimental models of inflammatory disease, autoimmunity and cancer. Scand J Immunol (2007) 66(2-3):143–58. 10.1111/j.1365-3083.2007.01986.x 17635792

[111] Gilson RC Gunasinghe SD Johannes L Gaus K. Galectin-3 modulation of T-cell activation: mechanisms of membrane remodelling. Prog Lipid Res (2019) 76:101010. 10.1016/j.plipres.2019.101010 31682868

[112] Smith LK Boukhaled GM Condotta SA Mazouz S Guthmiller JJ Vijay R. Interleukin-10 Directly Inhibits CD8+ T Cell Function by Enhancing N-Glycan Branching to Decrease Antigen Sensitivity. Immunity (2018) 48:299–312.e5. 10.1016/j.immuni.2018.01.006 29396160 PMC5935130

[113] Naing A Infante JR Papadopoulos KP Chan IH Shen C Ratti NP. PEGylated IL-10 (Pegilodecakin) Induces Systemic Immune Activation, CD8+ T Cell Invigoration and Polyclonal T Cell Expansion in Cancer Patients. Cancer Cell (2018) 34(5):775–91.e3. 10.1016/j.ccell.2018.10.007 30423297 PMC8098754

[114] Mumm JB Emmerich J Zhang X Chan I Wu L Mauze S. IL-10 Elicits IFNγ-Dependent tumor immune surveillance. Cancer Cell (2011) 20(6):781–96. 10.1016/j.ccr.2011.11.003 22172723

[115] Emmerich J Mumm JB Chan IH LaFace D Truong H McClanahan T. IL-10 directly activates and expands tumor-resident CD8+ T cells without De Novo infiltration from secondary lymphoid organs. Cancer Res (2012) 72(14):3570–81. 10.1158/0008-5472.CAN-12-0721 22581824

[116] Massagué J. TGFβ in Cancer. Cell (2008) 134(2):215–30. 10.1016/j.cell.2008.07.001 PMC3512574 18662538

[117] Yang L Moses HL. Transforming growth factor β: Tumor suppressor or promoter? Are host immune cells the answer? Cancer Res (2008) 68:9107–11. 10.1158/0008-5472.CAN-08-2556 PMC2741321 19010878

[118] Ng CT Snell LM Brooks DG Oldstone MBA. Networking at the level of host immunity: Immune cell interactions during persistent viral infections. Cell Host Microbe (2013) 13:652–64. 10.1016/j.chom.2013.05.014 PMC3713852 23768490

[119] Goh C Narayanan S Hahn YS. Myeloid-derived suppressor cells: The dark knight or the joker in viral infections? Immunol Rev (2013) 255:210–21. 10.1111/imr.12084 PMC3748397 23947357

[120] Waggoner SN Cornberg M Selin LK Welsh RM. Natural killer cells act as rheostats modulating antiviral T cells. Nature (2012) 481:394–8. 10.1038/nature10624 PMC3539796 22101430

[121] Holderried TAW Lang PA Kim HJ Cantor H. Genetic disruption of CD8+ Treg activity enhances the immune response to viral infection. Proc Natl Acad Sci USA (2013) 110:21089–94. 10.1073/pnas.1320999110 PMC3876206 24324159

[122] Veiga-Parga T Sehrawat S Rouse BT. Role of regulatory T cells during virus infection. Immunol Rev (2013) 255:182–96. 10.1111/imr.12085 PMC3748387 23947355

[123] Penaloza-MacMaster P Kamphorst AO Wieland A Araki K Iyer SS West EE. Interplay between regulatory T cells and PD-1 in modulating T cell exhaustion and viral control during chronic LCMV infection. J Exp Med (2014) 211:1905–18. 10.1084/jem.20132577 PMC4144726 25113973

[124] Wong EA Joslyn L Grant NL Klein E Lin PL Kirschner DE. Low levels of T cell exhaustion in tuberculous lung granulomas. Infect Immun (2018) 86:e00426–18. 10.1128/IAI.00426-18 PMC6105875 29891540

[125] Gigley JP Bhadra R Moretto MM Khan IA. T cell exhaustion in protozoan disease. Trends Parasitol (2012) 28:377–84. 10.1016/j.pt.2012.07.001 PMC3768288 22832368

[126] Yi JS Cox MA Zajac AJ. T-cell exhaustion: Characteristics, causes and conversion. Immunology (2010) 129:474–81. 10.1111/j.1365-2567.2010.03255.x PMC2842494 20201977

[127] Moskophidis D Battegay M Van den Broek M Laine E Hoffmann-Rohrer U Zinkernagel RM. Role of virus and host variables in virus persistence or immunopathological disease caused by a non-cytolytic virus. J Gen Virol (1995) 76:381–91. 10.1099/0022-1317-76-2-381 7531218

[128] Shankar P Russo M Harnisch B Patterson M Skolnik P Lieberman J. Impaired function of circulating HIV-specific CD8+ T cells in chronic human immunodeficiency virus infection. Blood (2000) 96:3094–101. 10.1182/blood.v96.9.3094 11049989

[129] Reignat S Webster GJM Brown D Ogg GS King A Seneviratne SL. Escaping high viral load exhaustion: CD8 cells with altered tetramer binding in chronic hepatitis B virus infection. J Exp Med (2002) 195:1089–101. 10.1084/jem.20011723 PMC2193712 11994415

[130] Sandu I Cerletti D Claassen M Oxenius A. Exhausted CD8+ T cells exhibit low and strongly inhibited TCR signaling during chronic LCMV infection. Nat Commun (2020) 11:4454. 10.1038/s41467-020-18256-4 32901001 PMC7479152

[131] Matloubian M Concepcion RJ Ahmed R. CD4+ T cells are required to sustain CD8+ cytotoxic T-cell responses during chronic viral infection. J Virol (1994) 68:8056–63. 10.1128/jvi.68.12.8056-8063.1994 PMC237269 7966595

[132] Thomsen AR Nansen A Andreasen SO Wodarz D Christensen JP. Host factors influencing viral persistence. Philos Trans R Soc B Biol Sci (2000) 355:1031–41. 10.1098/rstb.2000.0640 PMC1692806 11186304

[133] Sauce D Almeida JR Larsen M Haro L Autran B Freeman GJ. PD-1 expression on human CD8 T cells depends on both state of differentiation and activation status. AIDS (2007) 21:2005–13. 10.1097/QAD.0b013e3282eee548 17885290

[134] Agata Y Kawasaki A Nishimura H Ishida Y Tsubata T Yagita H. Expression of the PD-1 antigen on the surface of stimulated mouse T and B lymphocytes. Int Immunol (1996) 8:765–72. 10.1093/intimm/8.5.765 8671665

[135] Walunas TL Lenschow DJ Bakker CY Linsley PS Freeman GJ Green JM. CTLA-4 can function as a negative regulator of T cell activation. Immunity (1994) 1:405–13. 10.1016/1074-7613(94)90071-X 7882171

[136] Ahn E Araki K Hashimoto M Li W Riley JL Cheung J. Role of PD-1 during effector CD8 T cell differentiation. Proc Natl Acad Sci USA (2018) 115:4749–54. 10.1073/pnas.1718217115 PMC5939075 29654146

[137] Baitsch L Legat A Barba L Marraco SA Rivals JP Baumgaertner P. Extended co-expression of inhibitory receptors by human CD8 T-cells depending on differentiation, antigen-specificity and anatomical localization. PloS One (2012) 7:e30852. 10.1371/journal.pone.0030852 22347406 PMC3275569

[138] Duraiswamy J Ibegbu CC Masopust D Miller JD Araki K Doho GH. Phenotype, Function, and Gene Expression Profiles of Programmed Death-1 hi CD8 T Cells in Healthy Human Adults. J Immunol (2011) 186:4200–12. 10.4049/jimmunol.1001783 PMC3723805 21383243

[139] Keir ME Butte MJ Freeman GJ Sharpe AH. PD-1 and Its Ligands in Tolerance and Immunity. Annu Rev Immunol (2008) 26:677–704. 10.1146/annurev.immunol.26.021607.090331 18173375 PMC10637733

[140] Quigley M Pereyra F Nilsson B Porichis F Fonseca C Eichbaum Q. Transcriptional analysis of HIV-specific CD8+ T cells shows that PD-1 inhibits T cell function by upregulating BATF. Nat Med (2010) 16:1147–51. 10.1038/nm.2232 PMC3326577 20890291

[141] Zinselmeyer BH Heydari S Sacristán C Nayak D Cammer M Herz J. PD-1 promotes immune exhaustion by inducing antiviral T cell motility paralysis. J Exp Med (2013) 210:757–74. 10.1084/jem.20121416 PMC3620347 23530125

[142] Johnston RJ Comps-Agrar L Hackney J Yu X Huseni M Yang Y. The Immunoreceptor TIGIT Regulates Antitumor and Antiviral CD8+T Cell Effector Function. Cancer Cell (2014) 26:923–37. 10.1016/j.ccell.2014.10.018 25465800

[143] Workman CJ Dugger KJ Vignali DAA. Cutting Edge: Molecular Analysis of the Negative Regulatory Function of Lymphocyte Activation Gene-3. J Immunol (2002) 169:5392–5. 10.4049/jimmunol.169.10.5392 12421911

[144] Kuchroo VK Dardalhon V Xiao S Anderson AC. New roles for TIM family members in immune regulation. Nat Rev Immunol (2008) 8:577–80. 10.1038/nri2366 18617884

[145] Jin HT Anderson AC Tan WG West EE Ha SJ Araki K. Cooperation of Tim-3 and PD-1 in CD8 T-cell exhaustion during chronic viral infection. Proc Natl Acad Sci USA (2010) 107:14733–8. 10.1073/pnas.1009731107 PMC2930455 20679213

[146] Chen JY Feeney ER Chung RT. HCV and HIV co-infection: Mechanisms and management. Nat Rev Gastroenterol Hepatol (2014) 11:362–71. 10.1038/nrgastro.2014.17 PMC4330991 24535328

[147] Sansom DM. CD28, CTLA-4 and their ligands: Who does what and to whom? Immunology (2000) 101:169–77. 10.1046/j.1365-2567.2000.00121.x PMC2327073 11012769

[148] Qureshi OS Zheng Y Nakamura K Attridge K Manzotti C Schmidt EM. Trans-endocytosis of CD80 and CD86: A molecular basis for thecell-extrinsic function of CTLA-4. Science (2011) (80-):600–3. 10.1126/science.1202947 PMC3198051 21474713

[149] Matsuzaki J Gnjatic S Mhawech-Fauceglia P Beck A Miller A Tsuji T. Tumor-infiltrating NY-ESO-1-specific CD8+ T cells are negatively regulated by LAG-3 and PD-1 in human ovarian cancer. Proc Natl Acad Sci USA (2010) 107:7875–80. 10.1073/pnas.1003345107 PMC2867907 20385810

[150] Fourcade J Sun Z Benallaoua M Guillaume P Luescher IF Sander C. Upregulation of Tim-3 and PD-1 expression is associated with tumor antigen-specific CD8+ T cell dysfunction in melanoma patients. J Exp Med (2010) 72:917–27. 10.1084/jem.20100637 PMC2947081 20819923

[151] Woo SR Turnis ME Goldberg MV Bankoti J Selby M Nirschl CJ. Immune inhibitory molecules LAG-3 and PD-1 synergistically regulate T-cell function to promote tumoral immune escape. Cancer Res (2012) 121:2350–60. 10.1158/0008-5472.CAN-11-1620 PMC3288154 22186141

[152] Baitsch L Baumgaertner P Devêvre E Raghav SK Legat A Barba L. Exhaustion of tumor-specific CD8+ T cells in metastases from melanoma patients. J Clin Invest (2011) 6:209–17. 10.1172/JCI46102 PMC3104769 21555851

[153] Bianchi G Borgonovo G Pistoia V Raffaghello L. Immunosuppressive cells and tumour microenvironment: Focus on mesenchymal stem cells and myeloid derived suppressor cells. Histol Histopathol (2011) 26:941–51. 10.14670/HH-26.941 21630223

[154] Kouidhi S Elgaaied AB Chouaib S. Impact of Metabolism on T-Cell Differentiation and Function and Cross Talk with Tumor Microenvironment. Front Immunol (2017) 8:270. 10.3389/fimmu.2017.00270 28348562 PMC5346542

[155] Leone RD Powell JD. Metabolism of immune cells in cancer. Nat Rev Cancer (2020) 20:516–31. 10.1038/s41568-020-0273-y PMC8041116 32632251

[156] Kumar V Donthireddy L Marvel D Condamine T Wang F Lavilla-Alonso S. Cancer-Associated Fibroblasts Neutralize the Anti-tumor Effect of CSF1 Receptor Blockade by Inducing PMN-MDSC Infiltration of Tumors. Cancer Cell (2017) 32:654–68.e5. 10.1016/j.ccell.2017.10.005 29136508 PMC5827952

[157] Ugel S De Sanctis F Mandruzzato S Bronte V. Tumor-induced myeloid deviation: when myeloid-derived suppressor cells meet tumor-associated macrophages. J Clin Invest (2015) 125:3365–76. 10.1172/JCI80006 PMC4588310 26325033

[158] Jiang L Fang X Wang H Li D Wang X. Ovarian Cancer-Intrinsic Fatty Acid Synthase Prevents Anti-tumor Immunity by Disrupting Tumor-Infiltrating Dendritic Cells. Front Immunol (2018) 9:2927. 10.3389/fimmu.2018.02927 30619288 PMC6302125

[159] Gajewski TF Schreiber H Fu Y-X. Innate and adaptive immune cells in the tumor microenvironment. Nat Immunol (2013) 14:1014–22. 10.1038/ni.2703 PMC4118725 24048123

[160] Baitsch L Fuertes-Marraco SA Legat A Meyer C Speiser DE. The three main stumbling blocks for anticancer T cells. Trends Immunol (2012) 33(7):364–72. 10.1016/j.it.2012.02.006 22445288

[161] Riaz N Havel JJ Makarov V Desrichard A Urba WJ Sims JS. Tumor and Microenvironment Evolution during Immunotherapy with Nivolumab. Cell (2017) 171:934–49.e16. 10.1016/j.cell.2017.09.028 29033130 PMC5685550

[162] O’Donnell JS Teng MWL Smyth MJ. Cancer immunoediting and resistance to T cell-based immunotherapy. Nat Rev Clin Oncol (2019) 16:151–67. 10.1038/s41571-018-0142-8 30523282

[163] Dunn GP Old LJ Schreiber RD. The three Es of cancer immunoediting. Annu Rev Immunol (2004) 22:329–60. 10.1146/annurev.immunol.22.012703.104803 15032581

[164] Smyth MJ Dunn GP Schreiber RD. Cancer Immunosurveillance and Immunoediting: The Roles of Immunity in Suppressing Tumor Development and Shaping Tumor Immunogenicity. Adv Immunol (2006) 90:1–50. 10.1016/S0065-2776(06)90001-7 16730260

[165] Vesely MD Kershaw MH Schreiber RD Smyth MJ. Natural innate and adaptive immunity to cancer. Annu Rev Immunol (2011) 29:235–71. 10.1146/annurev-immunol-031210-101324 21219185

[166] Dunn GP Sheehan KCF Old LJ Schreiber RD. IFN unresponsiveness in LNCaP cells due to the lack of JAK1 gene expression. Cancer Res (2005) 65:3447–53. 10.1158/0008-5472.CAN-04-4316 15833880

[167] Takeda K Nakayama M Hayakawa Y Kojima Y Ikeda H Imai N. IFN-γ is required for cytotoxic T cell-dependent cancer genome immunoediting. Nat Commun (2017) 8:14607. 10.1038/ncomms14607 28233863 PMC5333095

[168] Mandai M Hamanishi J Abiko K Matsumura N Baba T Konishi I. Dual Faces of IFNγ in Cancer Progression: A Role of PD-L1 Induction in the Determination of Pro- and Antitumor Immunity. Clin Cancer Res an Off J Am Assoc Cancer Res (2016) 22:2329–34. 10.1158/1078-0432.CCR-16-0224 27016309

[169] Chang C-C Pirozzi G Wen S-H Chung I-H Chiu B-L Errico S. Multiple structural and epigenetic defects in the human leukocyte antigen class I antigen presentation pathway in a recurrent metastatic melanoma following immunotherapy. J Biol Chem (2015) 290:26562–75. 10.1074/jbc.M115.676130 PMC4646314 26381407

[170] Sharma P Hu-Lieskovan S Wargo JA Ribas A. Primary, Adaptive, and Acquired Resistance to Cancer Immunotherapy. Cell (2017) 168:707–23. 10.1016/j.cell.2017.01.017 PMC5391692 28187290

[171] Ochsenbein AF Klenerman P Karrer U Ludewig B Pericin M Hengartner H. Immune surveillance against a solid tumor fails because of immunological ignorance. Proc Natl Acad Sci USA (1999) 96:2233–8. 10.1073/pnas.96.5.2233 PMC26766 10051624

[172] Dustin ML. The immunological synapse. Cancer Immunol Res (2014) 2(11):1023–33. 10.1158/2326-6066.CIR-14-0161 PMC4692051 25367977

[173] Razvag Y Neve-Oz Y Sajman J Reches M Sherman E. Nanoscale kinetic segregation of TCR and CD45 in engaged microvilli facilitates early T cell activation. Nat Commun (2018) 9(1):732. 10.1038/s41467-018-03127-w 29467364 PMC5821895

[174] Jung Y Riven I Feigelson SW Kartvelishvily E Tohya K Miyasaka M. Three-dimensional localization of T-cell receptors in relation to microvilli using a combination of superresolution microscopies. Proc Natl Acad Sci USA (2016) 113(40):E5916–24. 10.1073/pnas.1605399113 27647916 PMC5056101

[175] Ghosh S Di Bartolo V Tubul L Shimoni E Kartvelishvily E Dadosh T. ERM-Dependent Assembly of T Cell Receptor Signaling and Co-stimulatory Molecules on Microvilli prior to Activation. Cell Rep (2020) 30(10):3434–47.e6. 10.1016/j.celrep.2020.02.069 32160548

[176] Welch MD Mullins RD. Cellular control of actin nucleation. Annu Rev Cell Dev Biol (2002) 18:247–88. 10.1146/annurev.cellbio.18.040202.112133 12142287

[177] Mattila PK Lappalainen P. Filopodia: Molecular architecture and cellular functions. Nat Rev Mol Cell Biol (2008) 9(6):446–54. 10.1038/nrm2406 18464790

[178] Kumari S Curado S Mayya V Dustin ML. T cell antigen receptor activation and actin cytoskeleton remodeling. Biochim Biophys Acta - Biomembr (2014) 1838(2):546–56. 10.1016/j.bbamem.2013.05.004 PMC3877165 23680625

[179] Husson J Chemin K Bohineust A Hivroz C Henry N. Force generation upon T cell receptor engagement. PloS One (2011) 6:e19680. 10.1371/journal.pone.0019680 21572959 PMC3091878

[180] Hui KL Balagopalan L Samelson LE Upadhyaya A. Cytoskeletal forces during signaling activation in Jurkat T-cells. Mol Biol Cell (2015) 26:685–95. 10.1091/mbc.E14-03-0830 PMC4325839 25518938

[181] Dustin ML. What counts in the immunological synapse? Mol Cell (2014) 54(2):255–62. 10.1016/j.molcel.2014.04.001 PMC4005017 24766889

[182] Dustin ML Long EO. Cytotoxic immunological synapses. Immunol Rev (2010) 235:24–34. 10.1111/j.0105-2896.2010.00904.x 20536553 PMC2950621

[183] Yokosuka T Sakata-Sogawa K Kobayashi W Hiroshima M Hashimoto-Tane A Tokunaga M. Newly generated T cell receptor microclusters initiate and sustain T cell activation by recruitment of Zap70 and SLP-76. Nat Immunol (2005) 6:1253–62. 10.1038/ni1272 16273097

[184] Davenport AJ Cross RS Watson KA Liao Y Shi W Prince HM. Chimeric antigen receptor T cells form nonclassical and potent immune synapses driving rapid cytotoxicity. Proc Natl Acad Sci USA (2018) 115:E2068–76. 10.1073/pnas.1716266115 29440406 PMC5834689

[185] Varma R Campi G Yokosuka T Saito T Dustin ML. T Cell Receptor-Proximal Signals Are Sustained in Peripheral Microclusters and Terminated in the Central Supramolecular Activation Cluster. Immunity (2006) 25:117–27. 10.1016/j.immuni.2006.04.010 PMC1626533 16860761

[186] Mariuzza RA Agnihotri P Orban J. The structural basis of T-cell receptor (TCR) activation: An enduring enigma. J Biol Chem (2020) 295:914–25. 10.1074/jbc.REV119.009411 PMC6983839 31848223

[187] Acuto O Cantrell D. T Cell Activation and the Cytoskeleton. Annu Rev Immunol (2000) 18:165–84. 10.1146/annurev.immunol.18.1.165 10837056

[188] Dupré L Houmadi R Tang C Rey-Barroso J. T lymphocyte migration: An action movie starring the actin and associated actors. Front Immunol (2015) 6:586. 10.3389/fimmu.2015.00586 26635800 PMC4649030

[189] Ksionda O Saveliev A Köchl R Rapley J Faroudi M Smith-Garvin JE. Mechanism and function of Vav1 localisation in TCR signalling. J Cell Sci (2012) 125(Pt 22):5302–14. 10.1242/jcs.105148 PMC3561853 22956543

[190] Helou YA Petrashen AP Salomon AR. Vav1 Regulates T-Cell Activation through a Feedback Mechanism and Crosstalk between the T-Cell Receptor and CD28. J Proteome Res (2015) 14(7):2963–75. 10.1021/acs.jproteome.5b00340 PMC4490005 26043137

[191] Reynolds LF Smyth LA Norton T Freshney N Downward J Kioussis D. Vav1 transduces T cell receptor signals to the activation ofphospholipase C-γ1 via phosphoinositide 3-kinase-dependent and -independentpathways. J Exp Med (2002) 195(9):1103–14. 10.1084/jem.20011663 PMC2193701 11994416

[192] Costello PS Walters AE Mee PJ Turner M Reynolds LF Prisco A. The Rho-family GTP exchange factor Vav is a critical transducer of Tcell receptor signals to the calcium, ERK, and NF-κB pathways. Proc Natl Acad Sci USA (1999) 96:3035–40. 10.1073/pnas.96.6.3035 PMC15890 10077632

[193] Zeng R Cannon JL Abraham RT Way M Billadeau DD Bubeck-Wardenberg J. SLP-76 Coordinates Nck-Dependent Wiskott-Aldrich Syndrome Protein Recruitment with Vav-1/Cdc42-Dependent Wiskott-Aldrich Syndrome Protein Activation at the T Cell-APCContact Site. J Immunol (2003) 171:1360–8. 10.4049/jimmunol.171.3.1360 12874226

[194] Zipfel PA Bunnell SC Witherow DS Gu JJ Chislock EM Ring C. Role for the Abi/Wave protein complex in T cell receptor-mediatedproliferation and cytoskeletal remodeling. Curr Biol (2006) 16:35–46. 10.1016/j.cub.2005.12.024 16401422

[195] Nolz JC Gomez TS Zhu P Li S Medeiros RB Shimizu Y. The WAVE2 complex regulates actin cytoskeletal reorganization andCRAC-mediated calcium entry during T cell activation. Curr Biol (2006) 16:24–34. 10.1016/j.cub.2005.11.036 16401421 PMC1779663

[196] Berke G. The CTL’s kiss of death. Cell (1995) 81:9–12. 10.1016/0092-8674(95)90365-8 7536631

[197] Anikeeva N Somersalo K Sims TN Thomas VK Dustin ML Sykulev Y. Distinct role of lymphocyte function-associated antigen-1 inmediating effective cytolytic activity by cytotoxic T lymphocytes. Proc Natl Acad Sci USA (2005) 102:6437–42. 10.1073/pnas.0502467102 PMC1088394 15851656

[198] Beal AM Anikeeva N Varma R Cameron TO Norris PJ Dustin ML. Protein Kinase Cθ Regulates Stability of the Peripheral Adhesion Ring Junction and Contributes to the Sensitivity of Target Cell Lysis by CTL. J Immunol (2008) 181:4815–24. 10.4049/jimmunol.181.7.4815 PMC2748977 18802085

[199] Mentlik AN Sanborn KB Holzbaur EL Orange JS. Rapid lytic granule convergence to the MTOC in natural killer cellsis dependent on dynein but not cytolytic commitment. Mol Biol Cell (2010) 21:2241–56. 10.1091/mbc.E09-11-0930 PMC2893988 20444980

[200] Pores-Fernando AT Zweifach A. Calcium influx and signaling in cytotoxic T-lymphocyte lytic granuleexocytosis. Immunol Rev (2009) 231:160–73. 10.1111/j.1600-065X.2009.00809.x 19754896

[201] Wiedemann A Depoil D Faroudi M Valitutti S. Cytotoxic T lymphocytes kill multiple targets simultaneously viaspatiotemporal uncoupling of lytic and stimulatory synapses. Proc Natl Acad Sci USA (2006) 103:10985–90. 10.1073/pnas.0600651103 PMC1544161 16832064

[202] Kearney CJ Brennan AJ Darcy PK Oliaro J. The role of the immunological synapse formed by cytotoxiclymphocytes in immunodeficiency and anti-tumor immunity. Crit Rev Immunol (2015) 35:325–47. 10.1615/CritRevImmunol.2015014417 26757394

[203] Kallikourdis M Viola A Benvenuti F. Human immunodeficiencies related to defective APC/T cellinteraction. Front Immunol (2015) 6:433. 10.3389/fimmu.2015.00433 26379669 PMC4551858

[204] Hanna S Etzioni A. Leukocyte adhesion deficiencies. Ann N Y Acad Sci (2012) 27:101–16. 10.1111/j.1749-6632.2011.06389.x 22276660

[205] Krensky AM Mentzer SJ Clayberger C Anderson DC Schmalstieg FC Burakoff SJ. Heritable lymphocyte function-associated antigen-1 deficiency: Abnormalities of cytotoxicity and proliferation associated with abnormal expression of LFA-1. J Immunol (1985) 135:3102–8. 3900204

[206] Orange JS Ramesh N Remold-O’Donnell E Sasahara Y Koopman L Byrne M. Wiskott-Aldrich syndrome protein is required for NK cellcytotoxicity and colocalizes with actin to NK cell-activating immunologic synapses. Proc Natl Acad Sci USA (2002) 99:11351–6. 10.1073/pnas.162376099 PMC123260 12177428

[207] Menotti M Ambrogio C Cheong TC Pighi C Mota I Cassel SH. Wiskott–Aldrich syndrome protein (WASP) is a tumor suppressorin T cell lymphoma. Nat Med (2019) 25:130–40. 10.1038/s41591-018-0262-9 PMC6556382 30510251

[208] Catucci M Zanoni I Draghici E Bosticardo M Castiello MC Venturini M. Wiskott-Aldrich syndrome protein deficiency in natural killer anddendritic cells affects antitumor immunity. Eur J Immunol (2014) 44:1039–45. 10.1002/eji.201343935 24338698

[209] Ishihara D Dovas A Hernandez L Pozzuto M Wyckoff J Segall JE. Wiskott-Aldrich syndrome protein regulates leukocyte-dependentbreast cancer metastasis. Cell Rep (2013) 4:429–36. 10.1016/j.celrep.2013.07.007 PMC3777703 23911287

[210] Kritikou JS Dahlberg CIM Baptista MAP Wagner AK Banerjee PP Gwalani LA. IL-2 in the tumor microenvironment is necessary for Wiskott-Aldrichsyndrome protein deficient NK cells to respond to tumors in vivo article. Sci Rep (2016) 6:30636. 10.1038/srep30636 27477778 PMC4967920

[211] Orange JS Roy-Ghanta S Mace EM Maru S Rak GD Sanborn KB. IL-2 induces a WAVE2-dependent pathway for actin reorganization thatenables WASp-independent human NK cell function. J Clin Invest (2011) 121:1535–48. 10.1172/JCI44862 PMC3069781 21383498

[212] Klevorn LE Berrien-Elliott MM Yuan J Kuehm LM Felock GD Crowe SA. Rescue of tolerant CD8+ T cells during cancer immunotherapy withIL2: Antibody complexes. Cancer Immunol Res (2016) 4:1016–26. 10.1158/2326-6066.CIR-16-0159 PMC5135645 27803062

[213] Moulding DA Record J Malinova D Thrasher AJ. Actin cytoskeletal defects in immunodeficiency. Immunol Rev (2013) 256:282–99. 10.1111/imr.12114 PMC3884764 24117828

[214] Dupont L Reeves MB. Cytomegalovirus latency and reactivation: recent insights into anage old problem. Rev Med Virol (2016) 26:75–89. 10.1002/rmv.1862 26572645 PMC5458136

[215] Brisse E Wouters CH Andrei G Matthys P. How viruses contribute to the pathogenesis of hemophagocyticlymphohistiocytosis. Front Immunol (2017) 8:1102. 10.3389/fimmu.2017.01102 28936212 PMC5594061

[216] Ramsay AG Clear AJ Kelly G Fatah R Matthews J MacDougall F. Follicular lymphoma cells induce T-cell immunologic synapsedysfunction that can be repaired with lenalidomide: Implications for the tumor microenvironment and immunotherapy. Blood (2009) 114:4713–20. 10.1182/blood-2009-04-217687 PMC2780306 19786615

[217] Ramsay AG Clear AJ Fatah R Gribben JG. Multiple inhibitory ligands induce impaired T-cell immunologicsynapse function in chronic lymphocytic leukemia that can be blocked with lenalidomide: Establishinga reversible immune evasion mechanism in human cancer. Blood (2012) 120(7):1412–21. 10.1182/blood-2012-02-411678 PMC3423779 22547582

[218] Koneru M Schaer D Monu N Ayala A Frey AB. Defective Proximal TCR Signaling Inhibits CD8 + Tumor-Infiltrating Lymphocyte Lytic Function. J Immunol (2005)174(4):1830–40. 10.4049/jimmunol.174.4.1830 15699109

[219] Monu N Frey AB. Suppression of proximal T cell receptor signaling and lytic functionin CD8+ tumor-infiltrating T cells. Cancer Res (2007) 67(23):11447–54. 10.1158/0008-5472.CAN-07-1441 PMC3712860 18056473

[220] Thoulouze MI Sol-Foulon N Blanchet F Dautry-Varsat A Schwartz O Alcover A. Human Immunodeficiency Virus Type-1 Infection Impairs the Formationof the Immunological Synapse. Immunity (2006) 24(5):547–61. 10.1016/j.immuni.2006.02.016 16713973

[221] Buffalo CZ Iwamoto Y Hurley JH Ren X. How HIV Nef Proteins Hijack Membrane Traffic To PromoteInfection. J Virol (2019) 93(24):e01322–19. 10.1128/jvi.01322-19 PMC6880166 31578291

[222] Haller C Rauch S Michel N Hannemann S Lehmann MJ Keppler OT. The HIV-1 pathogenicity factor Nef interferes with maturation ofstimulatory T-lymphocyte contacts by modulation of N-Wasp activity. J Biol Chem (2006) 281(28):19618–30. 10.1074/jbc.M513802200 16687395

[223] Ott M Emiliani S Van Lint C Herbein G Lovett J Chirmule N. Immune hyperactivation of HIV-1-infected T cells mediated by Tat andthe CD28 pathway. Science (1997) (80-):1481–5. 10.1126/science.275.5305.1481 9045614

[224] Haas A Zimmermann K Oxenius A. Antigen-Dependent and -Independent Mechanisms of T and B CellHyperactivation during Chronic HIV-1 Infection. J Virol (2011) 85(23):12102–13. 10.1128/jvi.05607-11 PMC3209369 21849433

[225] Fortin JF Barat C Beauséjour Y Barbeau B Tremblay MJ. Hyper-responsiveness to stimulation of human immunodeficiencyvirus-infected CD4+ T cells requires Nef and Tat virus gene products and results from higher NFAT, NF-κB, and AP-1 induction. J Biol Chem (2004) 279(38):39520–31. 10.1074/jbc.M407477200 15258149

[226] Manninen A Huotari P Hiipakka M Renkema GH Saksela K. Activation of NFAT-Dependent Gene Expression by Nef: Conservationamong Divergent Nef Alleles, Dependence on SH3 Binding and Membrane Association, and Cooperationwith Protein Kinase C-θ. J Virol (2001) 75(6):3034–7. 10.1128/jvi.75.6.3034-3037.2001 PMC115932 11222731

[227] Feng Y Reinherz EL Lang MJ. αβ T Cell Receptor Mechanosensing Forces out Serial Engagement. Trends Immunol (2018) 39:596–609. 10.1016/j.it.2018.05.005 30060805 PMC6154790

[228] Judokusumo E Tabdanov E Kumari S Dustin ML Kam LC. Mechanosensing in T Lymphocyte Activation. Biophys J (2012) 102:L5–7. 10.1016/j.bpj.2011.12.011 PMC3260692 22339876

[229] Orr AW Helmke BP Blackman BR Schwartz MA. Mechanisms of mechanotransduction. DevCell (2006) 10(1):11–20. 10.1016/j.devcel.2005.12.006 16399074

[230] Minguet S Swamy M Alarcón B Luescher IF Schamel WWA. Full Activation of the T Cell Receptor Requires Both Clustering and Conformational Changes at CD3. Immunity (2007) 26:43–54. 10.1016/j.immuni.2006.10.019 17188005

[231] Li L Guo X Shi X Li C Wu W Yan C. Ionic CD3–Lck interaction regulates the initiation of T-cell receptor signaling. Proc Natl Acad Sci (2017) 114:E5891–9. 10.1073/pnas.1701990114 PMC5530670 28659468

[232] Liu CSC Raychaudhuri D Paul B Chakrabarty Y Ghosh AR Rahaman O. Cutting Edge: Piezo1 Mechanosensors Optimize Human T Cell Activation. J Immunol (2018) 200:1255–60. 10.4049/jimmunol.1701118 29330322

[233] Malissen B Bongrand P. Early T cell activation: integrating biochemical, structural, and biophysical cues. Annu Rev Immunol (2015) 33:539–61. 10.1146/annurev-immunol-032414-112158 25861978

[234] Li Y-C Chen B-M Wu P-C Cheng T-L Kao L-S Tao M-H. Cutting Edge: Mechanical Forces Acting on T Cells Immobilized via the TCR Complex Can Trigger TCR Signaling. J Immunol (2010) 184:5959–63. 10.4049/jimmunol.0900775 20435924

[235] Chen W Zhu C. Mechanical regulation of T-cell functions. Immunol Rev (2013) 256:160–76. 10.1111/imr.12122 PMC3818107 24117820

[236] Kim ST Takeuchi K Sun Z-YJ Touma M Castro CE Fahmy A. The alphabeta T cell receptor is an anisotropic mechanosensor. J Biol Chem (2009) 284:31028–37. 10.1074/jbc.M109.052712 PMC2781503 19755427

[237] Liu B Chen W Evavold BD Zhu C. Accumulation of dynamic catch bonds between TCR and agonistpeptide-MHC triggers T cell signaling. Cell (2014)157(2):357–368. 10.1016/j.cell.2014.02.053 24725404 PMC4123688

[238] Hong J Persaud SP Horvath S Allen PM Evavold BD Zhu C. Force-Regulated In Situ TCR-Peptide-Bound MHC Class II Kinetics Determine Functions of CD4+ T Cells. J Immunol (2015) 195:3557–64. 10.4049/jimmunol.1501407 PMC4592802 26336148

[239] O’Connor RS Hao X Shen K Bashour K Akimova T Hancock WW. Substrate rigidity regulates human T cell activation and proliferation. J Immunol (2012) 189:1330–9. 10.4049/jimmunol.1102757 PMC3401283 22732590

[240] Saitakis M Dogniaux S Goudot C Bufi N Asnacios S Maurin M. Different TCR-induced T lymphocyte responses are potentiated by stiffness with variable sensitivity. Elife (2017) 6:e23190. 10.7554/eLife.23190 28594327 PMC5464771

[241] Jin W Tamzalit F Chaudhuri PK Black CT Huse M Kam LC. T cell activation and immune synapse organization respond to the microscale mechanics of structured surfaces. Proc Natl Acad Sci USA (2019) 116:19835–40. 10.1073/pnas.1906986116 PMC6778209 31527238

[242] Basu R Whitlock BM Husson J Le Floc’h A Jin W Oyler-Yaniv A. Cytotoxic T Cells Use Mechanical Force to Potentiate Target Cell Killing. Cell (2016) 165:100–10. 10.1016/j.cell.2016.01.021 PMC4808403 26924577

[243] Hickey JW Dong Y Chung JW Salathe SF Pruitt HC Li X. Engineering an Artificial T-Cell Stimulating Matrix for Immunotherapy. Adv Mater (2019) 31:e1807359. 10.1002/adma.201807359 30968468 PMC8601018

[244] Aramesh M Stoycheva D Raaz L Klotzsch E. Engineering T-cell activation for immunotherapy by mechanical forces. Curr Opin BioMed Eng (2019) 10:134–41. 10.1016/j.cobme.2019.05.004

[245] Dembo M Torney DC Saxman K Hammer D. The reaction-limited kinetics of membrane-to-surface adhesion and detachment. Proc R Soc London Ser B Biol Sci (1988) 234:55–83. 10.1098/rspb.1988.0038 2901109

[246] Lee C Lou J Wen K McKane M Eskin SG Ono S. Actin depolymerization under force is governed by lysine 113:glutamic acid 195-mediated catch-slip bonds. Proc Natl Acad Sci USA (2013) 110:5022–7. 10.1073/pnas.1218407110 PMC3612643 23460697

[247] Sundd P Pospieszalska MK Cheung LS-L Konstantopoulos K Ley K. Biomechanics of leukocyte rolling. Biorheology (2011) 48:1–35. 10.3233/BIR-2011-0579 21515934 PMC3103268

[248] Das DK Feng Y Mallis RJ Li X Keskin DB Hussey RE. Force-dependent transition in the T-cell receptor β-subunit allosterically regulates peptide discrimination and pMHC bond lifetime. Proc Natl Acad Sci USA (2015) 112:1517–22. 10.1073/pnas.1424829112 PMC4321250 25605925

[249] Kolawole EM Andargachew R Liu B Jacobs JR Evavold BD. 2D Kinetic Analysis of TCR and CD8 Coreceptor for LCMV GP33 Epitopes. Front Immunol (2018) 9:2348. 10.3389/fimmu.2018.02348 30374353 PMC6197077

[250] Sibener LV Fernandes RA Kolawole EM Carbone CB Liu F McAffee D. Isolation of a Structural Mechanism for Uncoupling T Cell Receptor Signaling from Peptide-MHC Binding. Cell (2018) 174:672–87.e27. 10.1016/j.cell.2018.06.017 30053426 PMC6140336

[251] Liu B Chen W Natarajan K Li Z Margulies DH Zhu C. The cellular environment regulates in situ kinetics of T-cell receptor interaction with peptide major histocompatibility complex. Eur J Immunol (2015) 45:2099–110. 10.1002/eji.201445358 PMC5642113 25944482

[252] Lee MS Glassman CR Deshpande NR Badgandi HB Parrish HL Uttamapinant C. A Mechanical Switch Couples T Cell Receptor Triggering to theCytoplasmic Juxtamembrane Regions of CD3ζζ. Immunity (2015) 43(2):227–39. 10.1016/j.immuni.2015.06.018 PMC4545397 26231119

[253] Van Der Merwe PA Dushek O. Mechanisms for T cell receptor triggering. Nat Rev Immunol (2011) 11(1):47–55. 10.1038/nri2887 21127503

[254] Chang VT Fernandes RA Ganzinger KA Lee SF Siebold C McColl J. Initiation of T cell signaling by CD45 segregation at “closecontacts.” Nat Immunol (2016) 17(5):574–82. 10.1038/ni.3392 PMC4839504 26998761

[255] James JR Vale RD. Biophysical mechanism of T-cell receptor triggering in areconstituted system. Nature (2012) 487(7405):647–9. 10.1038/nature11220 PMC3393772 22763440

[256] Higuchi A Ling QD Chang Y Hsu ST Umezawa A. Physical cues of biomaterials guide stem cell differentiationfate. Chem Rev (2013)113(5):3297–328. 10.1021/cr300426x 23391258

[257] Yang Y Wang K Gu X Leong KW. Biophysical Regulation of Cell Behavior—Cross Talk betweenSubstrate Stiffness and Nanotopography. Engineering(2017) 3(1):36–54. 10.1016/J.ENG.2017.01.014 29071164 PMC5653318

[258] Tee SY Fu J Chen CS Janmey PA. Cell shape and substrate rigidity both regulate cellstiffness. Biophys J (2011) 100(5):L25–7. 10.1016/j.bpj.2010.12.3744 PMC3043219 21354386

[259] Uffmann K Maderwald S Ajaj W Galban CG Mateiescu S Quick HH. In vivo elasticity measurements of extremity skeletal muscle with MRelastography. NMR BioMed (2004) 17(4):181–90. 10.1002/nbm.887 15229931

[260] Gasiorowski JZ Murphy CJ Nealey PF. Biophysical cues and cell behavior: The big impact of littlethings. Annu Rev BioMed Eng (2013) 15:155–76. 10.1146/annurev-bioeng-071811-150021 23862676

[261] Friedl P Alexander S. Cancer invasion and the microenvironment: Plasticity andreciprocity. Cell (2011)147(5):992–1009. 10.1016/j.cell.2011.11.016 22118458

[262] Tilghman RW Cowan CR Mih JD Koryakina Y Gioeli D Slack-Davis JK. Matrix rigidity regulates cancer cell growth and cellularphenotype. PloS One (2010) 5(9):e12905. 10.1371/journal.pone.0012905 20886123 PMC2944843

[263] Handorf AM Zhou Y Halanski MA Li WJ. Tissue stiffness dictates development, homeostasis, and diseaseprogression. Organogenesis (2015)11(1):1–15. 10.1080/15476278.2015.1019687 25915734 PMC4594591

[264] Wullkopf L West AKV Leijnse N Cox TR Madsen CD Oddershede LB. Cancer cells’ ability to mechanically adjust to extracellularmatrix stiffness correlates with their invasive potential. Mol Biol Cell (2018) 29(20):2378–85. 10.1091/mbc.E18-05-0319 PMC6233061 30091653

[265] Hynes RO. Integrins: Bidirectional, allosteric signalingmachines. Cell (2002)110(6):673–87. 10.1016/S0092-8674(02)00971-6 12297042

[266] Naba A Clauser KR Lamar JM Carr SA Hynes RO. Extracellular matrix signatures of human mammary carcinoma identifynovel metastasis promoters. Elife (2014) 3:e01308. 10.7554/eLife.01308 24618895 PMC3944437

[267] Henke E Nandigama R Ergün S. Extracellular Matrix in the Tumor Microenvironment and Its Impact onCancer Therapy. Front Mol Biosci (2020) 6:160. 10.3389/fmolb.2019.00160 32118030 PMC7025524

[268] Merika EE Syrigos KN Saif MW. Desmoplasia in pancreatic cancer. Can we fight it? Gastroenterol Res Pract (2012) 2012:781765. 10.1155/2012/781765 23125850 PMC3485537

[269] Walker RA. The complexities of breast cancer desmoplasia. Breast Cancer Res (2001) 3(3):143–5. 10.1186/bcr287 PMC138677 11305947

[270] Levental KR Yu H Kass L Lakins JN Egeblad M Erler JT. Matrix Crosslinking Forces Tumor Progression by Enhancing IntegrinSignaling. Cell (2009) 139(5):891–906. 10.1016/j.cell.2009.10.027 19931152 PMC2788004

[271] Hartmann N Giese NA Giese T Poschke I Offringa R Werner J. Prevailing role of contact guidance in intrastromal T-cell trappingin human pancreatic cancer. Clin Cancer Res (2014) 20(13):3422–33. 10.1158/1078-0432.CCR-13-2972 24763614

[272] Salmon H Franciszkiewicz K Damotte D Dieu-Nosjean MC Validire P Trautmann A. Matrix architecture defines the preferential localization andmigration of T cells into the stroma of human lung tumors. J Clin Invest (2012) 122(3):899–910. 10.1172/JCI45817 22293174 PMC3287213

[273] Kuczek DE Larsen AMH Thorseth ML Carretta M Kalvisa A Siersbæk MS. Collagen density regulates the activity of tumor-infiltrating Tcells. J Immunother Cancer (2019) 7(1):68. 10.1186/s40425-019-0556-6 30867051 PMC6417085

[274] Han YL Pegoraro AF Li H Li K Yuan Y Xu G. Cell swelling, softening and invasion in a three-dimensional breastcancer model. Nat Phys (2020) 16(1):101–8. 10.1038/s41567-019-0680-8 PMC7469976 32905405

[275] Stone JD Chervin AS Kranz DM. T-cell receptor binding affinities and kinetics: impact on T-cellactivity and specificity. Immunology (2009) 126(2):165–76. 10.1111/j.1365-2567.2008.03015.x PMC2632691 19125887

[276] Zhong S Malecek K Johnson LA Yu Z De Miera EVS Darvishian F. T-cell receptor affinity and avidity defines antitumor response andautoimmunity in T-cell immunotherapy. Proc Natl Acad Sci USA (2013) 110(17):6973–8. 10.1073/pnas.1221609110 PMC3637771 23576742

[277] Hu Z Zhu L Wang J Wan Y Yuan S Chen J. Immune Signature of Enhanced Functional Avidity CD8+ T Cells in vivoInduced by Vaccinia Vectored Vaccine. Sci Rep (2017) 7:41558. 10.1038/srep41558 28155878 PMC5290741

[278] Turner SJ Doherty PC McCluskey J Rossjohn J. Structural determinants of T-cell receptor bias inimmunity. Nat Rev Immunol (2006) 6(12):883–94. 10.1038/nri1977 17110956

[279] Sabatino JJ Huang J Zhu C Evavold BD. High prevalence of low affinity peptide-MHC II tetramer-negativeeffectors during polyclonal CD4+ T cell responses. J Exp Med (2011) 208(1):81–90. 10.1084/jem.20101574 21220453 PMC3023139

[280] Martinez RJ Andargachew R Martinez HA Evavold BD. Low-affinity CD4+ T cells are major responders in the primary immuneresponse. Nat Commun (2016) 7:13848. 10.1038/ncomms13848 27976744 PMC5234832

[281] Savage PA Boniface JJ Davis MM. A kinetic basis for T cell receptor repertoire selection during animmune response. Immunity (1999) 10(4):485–92. 10.1016/S1074-7613(00)80048-5 10229191

[282] Malherbe L Hausl C Teyton L McHeyzer-Williams MG. Clonal selection of helper T cells is determined by an affinitythreshold with no further skewing of TCR binding properties. Immunity (2004) 21(5):6697–79. 10.1016/j.immuni.2004.09.008 15539153

[283] Busch DH Pamer EG. T cell affinity maturation by selective expansion duringinfection. J Exp Med (1999) 189(4):701–10. 10.1084/jem.189.4.701 PMC2192934 9989985

[284] Kedl RM Kappler JW Marrack P. Epitope dominance, competition and T cell affinitymaturation. Curr Opin Immunol (2003) 15(1):1207–7. 10.1016/S0952-7915(02)00009-2 12495743

[285] Lichterfeld M Yu XG Mui SK Williams KL Trocha A Brockman MA. Selective Depletion of High-Avidity Human Immunodeficiency VirusType 1 (HIV-1)-Specific CD8+ T Cells after Early HIV-1 Infection. J Virol (2007) 81(8):4199–214. 10.1128/jvi.01388-06 PMC1866095 17287271

[286] Rossjohn J Gras S Miles JJ Turner SJ Godfrey DI McCluskey J. T Cell Antigen Receptor Recognition of Antigen-PresentingMolecules. Annu Rev Immunol (2015) 33:169–200. 10.1146/annurev-immunol-032414-112334 25493333

[287] Huang J Zarnitsyna VI Liu B Edwards LJ Jiang N Evavold BD. The kinetics of two-dimensional TCR and pMHC interactions determineT-cell responsiveness. Nature (2010) 44(1):239–50. 10.1038/nature08944 PMC2925443 20357766

[288] Liu B Zhong S Malecek K Johnson LA Rosenberg SA Zhu C. 2D TCR-pMHC-CD8 kinetics determines T-cell responses in a self-antigen-specific TCR system. Eur J Immunol (2014). 10.1002/eji.201343774 PMC3941036 24114747

[289] Dolton G Zervoudi E Rius C Wall A Thomas HL Fuller A. Optimized peptide-MHC multimer protocols for detection and isolationof autoimmune T-cells. Front Immunol (2018) 9:1378. 10.3389/fimmu.2018.01378 30008714 PMC6034003

[290] Corr M Slanetz AE Boyd LF Jelonek MT Khilko S Al-Ramadi BK. T cell receptor-MHC class I peptide interactions: Affinity,kinetics, and specificity. Science (1994) 80-):946–9. 10.1126/science.8052850 8052850

[291] Margulies DH Plaksin D Khilko SN Jelonek MT. Studying interactions involving the T-cell antigen receptor bysurface plasmon resonance. Curr Opin Immunol (1996) 8(2):262–70. 10.1016/S0952-7915(96)80066-5 8725950

[292] Myszka DG. Improving biosensor analysis. J MolRecognit (1999) 12(5):279–84. 10.1002/(SICI)1099-1352(199909/10)12:5<279::AID-JMR473>3.0.CO;2-3 10556875

[293] Rich RL Myszka DG. Survey of the 2009 commercial optical biosensorliterature. J Mol Recognit (2011) 24(6):892–914. 10.1002/jmr.1138 22038797

[294] Chesla SE Selvaraj P Zhu C. Measuring two-dimensional receptor-ligand binding kinetics bymicropipette. Biophys J (1998) 75(3):1553–72. 10.1016/S0006-3495(98)74074-3 PMC1299830 9726957

[295] Boniface JJ Rabinowitz JD Wülfing C Hampl J Reich Z Altman JD. Initiation of signal transduction through the T cell receptorrequires the peptide multivalent engagement of MHC ligands. Immunity (1998) 9(4):459–66. 10.1016/S1074-7613(00)80629-9 9806632

[296] Bakker AH Schumacher TNM. MHC multimer technology: Current status and futureprospects. Curr Opin Immunol (2005) 17(4):428–33. 10.1016/j.coi.2005.06.008 15967654

[297] Altman JD Moss PAH Goulder PJR Barouch DH McHeyzer-Williams MG Bell JI. Phenotypic analysis of antigen-specific Tlymphocytes. Science (1996) (80-):94–6. 10.1126/science.274.5284.94 8810254

[298] Rius C Attaf M Tungatt K Bianchi V Legut M Bovay A. Peptide–MHC Class I Tetramers Can Fail To Detect RelevantFunctional T Cell Clonotypes and Underestimate Antigen-Reactive T Cell Populations. J Immunol (2018) 200(7):2263–2279. 10.4049/jimmunol.1700242 29483360 PMC5857646

[299] Al-Ramadi BK Jelonek MT Boyd LF Margulies DH Bothwell ALM. Lack of strict correlation of functional sensitization with theapparent affinity of MHC/peptide complexes for the TCR. J Immunol (1995) 155(2):662–73. 7541822

[300] Dougan SK Dougan M Kim J Turner JA Ogata S Il CH. Transnuclear TRP1-specific CD8 T cells with high or low affinityTCRs show equivalent antitumor activity. Cancer Immunol Res (2013) 1(2):99–111. 10.1158/2326-6066.CIR-13-0047 24459675 PMC3895912

[301] Cukalac T Chadderton J Handel A Doherty PC Turner SJ Thomas PG. Reproducible selection of high avidity CD8+ T-cell clones following secondary acute virus infection. Proc Natl Acad Sci USA (2014) 111(4):1485–90. 10.1073/pnas.1323736111 PMC3910643 24474775

[302] Zehn D Lee SY Bevan MJ. Complete but curtailed T-cell response to very low-affinityantigen. Nature (2009) 458(7235):211–4. 10.1038/nature07657 PMC2735344 19182777

[303] Krummey SM Martinez RJ Andargachew R Liu D Wagener M Kohlmeier JE. Low-Affinity Memory CD8 + T Cells Mediate Robust HeterologousImmunity. J Immunol (2016) 196(6):2838–46. 10.4049/jimmunol.1500639 PMC4779681 26864034

[304] Martinez RJ Evavold BD. Lower affinity T cells are critical components and activeparticipants of the immune response. Front Immunol (2015) 6:468. 10.3389/fimmu.2015.00468 26441973 PMC4564719

[305] Caserta S Kleczkowska J Mondino A Zamoyska R. Reduced Functional Avidity Promotes Central and Effector Memory CD4T Cell Responses to Tumor-Associated Antigens. J Immunol (2010) 185(11):6545–54. 10.4049/jimmunol.1001867 21048115

[306] Gallegos AM Xiong H Leiner IM Sušac B Glickman MS Pamer EG. Control of T cell antigen reactivity via programmed TCR downregulation. Nat Immunol (2016) 17(4):379–86. 10.1038/ni.3386 PMC4803589 26901151

[307] Rosenthal KM Edwards LJ Sabatino JJ Hood JD Wasserman HA Zhu C. Low 2-dimensional CD4 T cell receptor affinity for myelin sets inmotion delayed response kinetics. PloS One (2012) 7(3):e32562. 10.1371/journal.pone.0032562 22412888 PMC3296730

[308] Andargachew R Martinez RJ Kolawole EM Evavold BD. CD4 T Cell Affinity Diversity Is Equally Maintained during Acute andChronic Infection. J Immunol (2018) 201(1):19–30. 10.4049/jimmunol.1800295 29777029 PMC6497530

[309] Welten SPM Baumann NS Oxenius A. Fuel and brake of memory T cell inflation. Med Microbiol Immunol (2019) 208(3-4):329–38. 10.1007/s00430-019-00587-9 30852648

[310] Nauerth M Weißbrich B Knall R Franz T Dössinger G Bet J. TCR-ligand koff rate correlates with the protective capacity ofantigen-specific CD8+ T cells for adoptive transfer. Sci Transl Med (2013) 5(192):192ra87. 10.1126/scitranslmed.3005958 PMC3991308 23825303

[311] Davenport MP Fazou C McMichael AJ Callan MFC. Clonal Selection, Clonal Senescence, and Clonal Succession: TheEvolution of the T Cell Response to Infection with a Persistent Virus. JImmunol (2002) 168(7):3309–17. 10.4049/jimmunol.168.7.3309 11907087

[312] Viganò S Utzschneider DT Perreau M Pantaleo G Zehn D Harari A. Functional avidity: A measure to predict the efficacy of effector Tcells? Clin Dev Immunol (2012) 2012:153863. 10.1155/2012/153863 23227083 PMC3511839

[313] Utzschneider DT Alfei F Roelli P Barras D Chennupati V Darbre S. High antigen levels induce an exhausted phenotype in a chronicinfection without impairing T cell expansion and survival. J Exp Med (2016) 213(9):1819–34. 10.1084/jem.20150598 PMC4995073 27455951

[314] Miller AM Bahmanof M Zehn D Cohen EEW Schoenberger SP. Leveraging TCR affinity in adoptive immunotherapy against sharedtumor/self-antigens. Cancer Immunol Res (2019) 7(1):40–49. 10.1158/2326-6066.CIR-18-0371 30482746 PMC7793606

[315] Hoffmann MM Slansky JE. T-cell receptor affinity in the age of cancerimmunotherapy. Mol Carcinog (2020) 59(7):862–870. 10.1002/mc.23212 32386086 PMC7340130

[316] Bos R Marquardt KL Cheung J Sherman LA. Functional differences between low- and highaffinity CD8+ T cells inthe tumor environment. Oncoimmunology (2012) 1(8):1239–47. 10.4161/onci.21285 PMC3518496 23243587

[317] Shakiba M Philip M Camara S Socci ND Schietinger A. The impact of TCR affinity on T cell differentiation and dysfunctionin tumors. J Immunol (2018) 200(1 Supplement) 57.24.

[318] Thaxton JE Li Z. To affinity and beyond: Harnessing the T cell receptor for cancerimmunotherapy. Hum Vaccines Immunother (2014) 10(11):3313–21. 10.4161/21645515.2014.973314 PMC4514023 25483644

[319] Beauchemin L Slifker M Rossell D Font-Burgada J. Characterizing MHC-I Genotype Predictive Power for Oncogenic Mutation Probability in Cancer Patients. Methods Mol Biol (2020) 2131:185–98. 10.1007/978-1-0716-0389-5_8 32162254

[320] Mazzocco M Martini M Rosato A Stefani E Matucci A Dalla Santa S. Autologous cellular vaccine overcomes cancer immunoediting in a mouse model of myeloma. Immunology (2015) 146:33–49. 10.1111/imm.12477 25959091 PMC4552499

[321] Qamra A Xing M Padmanabhan N Kwok JJT Zhang S Xu C. Epigenomic Promoter Alterations Amplify Gene Isoform and Immunogenic Diversity in Gastric Adenocarcinoma. Cancer Discovery (2017) 7:630–51. 10.1158/2159-8290.CD-16-1022 28320776

[322] van Gisbergen KPJM Klarenbeek PL Kragten NAM Unger PPA Nieuwenhuis MBB Wensveen FM. The costimulatory molecule CD27 maintains clonally diverse CD8+ Tcell responses of low antigen affinity to protect against viral variants. Immunity (2011) 35(1):97–108. 10.1016/j.immuni.2011.04.020 21763160

[323] Oh S Hodge JW Ahlers JD Burke DS Schlom J Berzofsky JA. Selective Induction of High Avidity CTL by Altering the Balance ofSignals from APC. J Immunol (2003) 170(5):2523–30. 10.4049/jimmunol.170.5.2523 12594278

[324] Hodge JW Chakraborty M Kudo-Saito C Garnett CT Schlom J. Multiple Costimulatory Modalities Enhance CTLAvidity. J Immunol (2005) 174(10):5994–6004. 10.4049/jimmunol.174.10.5994 15879092 PMC1924685

[325] Viola A Lanzavecchia A. T cell activation determined by T cell receptor number and tunablethresholds. Science (1996) (80-):104–6. 10.1126/science.273.5271.104 8658175

[326] Bullock TNJ Mullins DW Engelhard VH. Antigen Density Presented By Dendritic Cells In Vivo DifferentiallyAffects the Number and Avidity of Primary, Memory, and Recall CD8 + T Cells. J Immunol (2003) 170(4):1822–9. 10.4049/jimmunol.170.4.1822 12574347

[327] Merkenschlager J Ploquin MJ Eksmond U Andargachew R Thorborn G Filby A. Stepwise B-cell-dependent expansion of T helper clonotypesdiversifies the T-cell response. Nat Commun (2016) 7:10281. 10.1038/ncomms10281 26728651 PMC4728444

[328] Mamula MJ Janeway CA. Do B cells drive the diversification of immuneresponses? Immunol Today (1993) 14(4):151–2. 10.1016/0167-5699(93)90274-O 8098941

[329] Iskratsch T Wolfenson H Sheetz MP. Appreciating force and shape-the rise of mechanotransduction in cellbiology. Nat Rev Mol Cell Biol (2014) 15(12):825–33. 10.1038/nrm3903 PMC9339222 25355507

[330] Colin-York H Javanmardi Y Skamrahl M Kumari S Chang VT Khuon S. Cytoskeletal Control of Antigen-Dependent T CellActivation. Cell Rep (2019) 26(12):3369–79.e5. 10.1016/j.celrep.2019.02.074 30893608 PMC6426652

[331] Bashoura KT Gondarenko A Chen H Shen K Liu X Huse M. CD28 and CD3 have complementary roles in T-cell tractionforces. Proc Natl Acad Sci USA (2014) 111(6):2241–6. 10.1073/pnas.1315606111 PMC3926067 24469820

[332] Liu Y Blanchfield L Pui-Yan Ma V Andargachew R Galior K Liu Z. DNA-based nanoparticle tension sensors reveal that T-cell receptorstransmit defined pN forces to their antigens for enhanced fidelity. ProcNatl Acad Sci USA (2016) 113(20):5610–5. 10.1073/pnas.1600163113 PMC4878516 27140637

[333] Ma VPY Liu Y Blanchfield L Su H Evavold BD Salaita K. Ratiometric tension probes for mapping receptor forces andclustering at intermembrane junctions. Nano Lett (2016) 16(7):4552–9. 10.1021/acs.nanolett.6b01817 PMC6061938 27192323

[334] Grupp SA Kalos M Barrett D Aplenc R Porter DL Rheingold SR. Chimeric antigen receptor-modified T cells for acute lymphoidleukemia. N Engl J Med (2013) 368(16):1509–18. 10.1056/NEJMoa1215134 PMC4058440 23527958

[335] Maude SL Teachey DT Porter DL Grupp SA. CD19-targeted chimeric antigen receptor T-cell therapy for acutelymphoblastic leukemia. Blood (2015) 125(26):4017–23. 10.1182/blood-2014-12-580068 PMC4481592 25999455

[336] June CH O’Connor RS Kawalekar OU Ghassemi S Milone MC. CAR T cell immunotherapy for human cancer. Science (2018) (80-):1361–65. 10.1126/science.aar6711 29567707

[337] Long AH Haso WM Shern JF Wanhainen KM Murgai M Ingaramo M. 4-1BB costimulation ameliorates T cell exhaustion induced by tonicsignaling of chimeric antigen receptors. Nat Med (2015) 21(6):581–90. 10.1038/nm.3838 PMC4458184 25939063

[338] Fraietta JA Lacey SF Orlando EJ Pruteanu-Malinici I Gohil M Lundh S. Determinants of response and resistance to CD19 chimeric antigenreceptor (CAR) T cell therapy of chronic lymphocytic leukemia. Nat Med (2018) 24(5):563–71. 10.1038/s41591-018-0010-1 PMC6117613 29713085

[339] Watanabe K Kuramitsu S Posey AD June CH. Expanding the therapeutic window for CAR T cell therapy in solidtumors: The knowns and unknowns of CAR T cell biology. Front Immunol (2018) 9:2486. 10.3389/fimmu.2018.02486 30416506 PMC6212550

